# MK-BMC: a Multi-Kernel framework with Boosted distance metrics for Microbiome data for Classification

**DOI:** 10.1093/bioinformatics/btad757

**Published:** 2024-01-10

**Authors:** Huang Xu, Tian Wang, Yuqi Miao, Min Qian, Yaning Yang, Shuang Wang

**Affiliations:** Department of Statistics and Finance, University of Science and Technology of China, Hefei 230026, China; Department of Biostatistics, Mailman School of Public Health, Columbia University, New York, NY 10032, United States; Department of Biostatistics, Mailman School of Public Health, Columbia University, New York, NY 10032, United States; Department of Biostatistics, Mailman School of Public Health, Columbia University, New York, NY 10032, United States; Department of Statistics and Finance, University of Science and Technology of China, Hefei 230026, China; Department of Biostatistics, Mailman School of Public Health, Columbia University, New York, NY 10032, United States

## Abstract

**Motivation:**

Research on human microbiome has suggested associations with human health, opening opportunities to predict health outcomes using microbiome. Studies have also suggested that diverse forms of taxa such as rare taxa that are evolutionally related and abundant taxa that are evolutionally unrelated could be associated with or predictive of a health outcome. Although prediction models were developed for microbiome data, no prediction models currently exist that use multiple forms of microbiome–outcome associations.

**Results:**

We developed MK-BMC, a Multi-Kernel framework with Boosted distance Metrics for Classification using microbiome data. We propose to first boost widely used distance metrics for microbiome data using taxon-level association signal strengths to up-weight taxa that are potentially associated with an outcome of interest. We then propose a multi-kernel prediction model with one kernel capturing one form of association between taxa and the outcome, where a kernel measures similarities of microbiome compositions between pairs of samples being transformed from a proposed boosted distance metric. We demonstrated superior prediction performance of (i) boosted distance metrics for microbiome data over original ones and (ii) MK-BMC over competing methods through extensive simulations. We applied MK-BMC to predict thyroid, obesity, and inflammatory bowel disease status using gut microbiome data from the American Gut Project and observed much-improved prediction performance over that of competing methods. The learned kernel weights help us understand contributions of individual microbiome signal forms nicely.

**Availability and implementation:**

Source code together with a sample input dataset is available at https://github.com/HXu06/MK-BMC

## 1 Introduction

The human microbiome is the collection of microorganisms including bacteria, viruses, archaea, and fungi living in the human body. The development of high-throughput sequencing technology has enabled efficient and detailed characterizations of microbial communities, leading to an explosive growth in studies investigating the human microbiome. There are two major sequencing approaches to quantify the composition of species. One is gene-targeted sequencing, where specific marker genes such as the 16S ribosomal RNA (rRNA) genes are amplified and sequenced (Tringe and Rubin 2005, Caporaso *et al.* 2010, Lasken 2012, Rapin *et al.* 2017). Sequencing reads are usually clustered into operational taxonomic units (OTUs) with a sequence similarity, such as 97% (Nguyen *et al.* 2016). A phylogenetic tree that captures evolutionary relationships among species can be constructed based on sequence divergences of OTUs (Price *et al.* 2010). Thus, OTUs that are close to a phylogenetic tree are usually also functionally related. The other method is the shotgun metagenomic sequencing, which sequences all microbial genomic DNA (Truong *et al.* 2015, Scholz *et al.* 2016). Although shotgun metagenomics can profile microbial communities more accurately, the targeted approach is more popular due to its low cost. Large studies such as the Human Microbiome Project (Consortium *et al.* 2012) and the American Gut Project (AGP, McDonald *et al.* 2018) used the targeted sequencing approach to generate microbiome data.

Over the past decades, studies have established associations between microbiome and health outcomes (Morgan *et al.* 2012, Wu *et al.* 2016b). Different statistical methods have been developed for microbiome data, and many of them used distance-based methods (Zhao *et al.* 2015, Wu *et al.* 2016a, Koh *et al.* 2017, Ma *et al.* 2020, Wang *et al.* 2022). The performance of distance-based methods is known to be greatly affected by distance metrics used (Chen *et al.* 2012). For microbiome data, several distance metrics have been developed and widely used. UniFrac distances (Lozupone and Knight 2005, Lozupone *et al.* 2007) weight branch lengths in a phylogenetic tree either by differences in the presence/absence of the descending OTUs between two samples, thus capturing signals of rare taxa (unweighted UniFrac distance) (Lozupone and Knight 2005), or by differences in abundance levels of the descending OTUs, thus capturing signals of abundance taxa (weighted UniFrac distance) (Lozupone *et al.* 2007). Generalized UniFrac distances (Chen *et al.* 2012) focus on OTUs in between. Another commonly used distance metric for microbiome data is the Bray–Curtis distance (Bray and Curtis 1957), which only considers abundance information of OTUs. Several existing methods that test for associations between microbiome and health outcomes consider multiple distance metrics (Zhao *et al.* 2015, Wu *et al.* 2016a, Koh *et al.* 2017) and choose an optimal one. That is, only one form of association between taxa and health outcomes is considered in the final model.

Studies have also investigated how microbiome predicts health outcomes using either general-purpose prediction methods such as Random Forest (Breiman 2001), sparse regression models like Lasso (Tibshirani 1996, Knights *et al.* 2011), or methods specifically developed for microbiome data for prediction (Tanaseichuk *et al.* 2014, Chen *et al.* 2015, Xiao *et al.* 2018, Wassan *et al.* 2018). Most recently, prediction models with deep learning methods were also developed using microbiome data (Grazioli *et al.* 2022, Wang *et al.* 2021, Sharma *et al.* 2020, Reiman *et al.* 2020) with many of them using a convolutional neural network (CNN) that can capture the spatial relationship. In these models, convolutional layers were used to mimic taxonomic ranks to capture the phylogenetic relationship among microbial species.

However, many studies have suggested that in real microbiome studies, multiple forms of microbiome–outcome association exist (Giliberti *et al.* 2022). For example, health outcomes including obesity (Turnbaugh *et al.* 2009), irritable bowel disease (Morgan *et al.* 2012), and diabetes (Karlsson *et al.* 2013) are associated with the presence/absence information of some taxa and are also associated with the abundance level of other taxa. In addition, the associated taxa may be close to each other on a phylogenetic tree (referred to as phylogenetically related) or are scattered on a phylogenetic tree (referred to as phylogenetically unrelated). For prediction purposes, no methods exist that consider multiple forms of microbiome–outcome associations.

In this paper, we propose MK-BMC, a Multi-Kernel framework with Boosted distance Metrics for Classifications using microbiome data, with each kernel being transformed from a boosted distance metric for microbiome data capturing one form of association between taxa and a health outcome. MK-BMC learns kernel weights for multiple kernels with kernel weights reflecting contributions of individual kernels, i.e. individual types of microbiome–outcome associations. Here we propose to first boost existing distance metrics for microbiome data using taxon-level association signal strength to up-weight taxa that are potentially associated with a health outcome of interest, and down-weight those that are potentially noises to further improve prediction. The proposed MK-BMC method then uses kernels derived from these boosted distance metrics. Through extensive simulation studies, we demonstrated the superior prediction performance of (i) the proposed boosted distance metrics over the original ones and (ii) the proposed MK-BMC method over several competing methods. We applied MK-BMC and competing methods to predict thyroid, obesity, and inflammatory bowel disease (IBD) status using gut microbiome data from the American Gut Project and observed much-improved prediction performance of MK-BMC over that of competing methods. The estimated kernel weights give insights into contributions of different forms of microbiome–outcome associations.

## 2 Methods

Let $y_{i}$ be the case–control status (1 for case, 0 for control), $P_{i}=(P_{i1},P_{i2},\ldots ,P_{iq})$ be the relative abundances levels of *q* OTUs, and $X_{i}=(X_{i1},X_{i2},\ldots ,X_{iL})$ be the *L* covariates (e.g. age, gender) for sample *i*, i=1,2,…,N. We denote T as the rooted phylogenetic tree with *R* branches with branch lengths $b_{1},b_{2},\ldots ,b_{R}$.

### 2.1 The proposed boosted distances for microbiome data

#### 2.1.1 Recap of distance metrics for microbiome data

Several popular distance metrics for microbiome data have been proposed (Kuczynski *et al.* 2010, Fukuyama *et al.* 2012, Tang *et al.* 2016). They can be categorized into tree-based distances, such as unweighted and weighted UniFrac distances (Lozupone and Knight 2005, Lozupone *et al.* 2007) calculated based on phylogenetic tree information, and non-tree-based distances including Bray–Curtis (Bray and Curtis 1957) and Hamming distances (Zhang *et al.* 2018) without incorporating phylogenetic tree information. Alternatively, these distances can be divided into abundance-based (using species’ abundance levels) and presence-absence-based (using species’ presence–absence status, Tang *et al.* 2016, Zhang *et al.* 2018). Specifically, the weighted UniFrac distance between samples *i* and *j* is defined as $d_{w}(i,j)=\frac{\sum_{r=1}^{R}b_{r}\left|p_{ir}-p_{jr}\right|}{\sum_{r=1}^{R}b_{r}\left(p_{ir}+p_{jr}\right)}$ for *R* branches. The unweighted UniFrac distance is defined as $d_{un}(i,j)=\frac{\sum_{r=1}^{R}b_{r}\left|I\left(p_{ir}>0\right)-I\left(p_{jr}>0\right)\right|}{\sum_{r=1}^{R}b_{r}I\left(p_{ir}+p_{jr}>0\right)}$, where I(⋅) is an indicator function. Both the Bray–Curtis distance $d_{BC}(i,j)=\frac{\sum_{r=1}^{q}\left|p_{ir}-p_{jr}\right|}{\sum_{r=1}^{q}\left(p_{ir}+p_{jr}\right)}=\frac{\sum_{r=1}^{q}\left|p_{ir}-p_{jr}\right|}{2}$ and the Hamming distance $d_{H}(i,j)=\sum_{r=1}^{q}\left|I\left(p_{ir}>0\right)-I\left(p_{jr}>0\right)\right|$ are calculated from the abundance levels of *q* OTUs without referring to phylogenetic tree information. Note that the Hamming distance is equivalent to the presence–absence version of the Bray–Curtis distance, as the denominator of the Bray–Curtis distance is actually a constant.

#### 2.1.2 The proposed boosted distance metrics for microbiome data

The aforementioned four distance metrics comprehensively quantify the difference in microbiome compositions between two samples. However, to predict health outcomes using microbiome, not all taxa in the entire microbiome of a sample are predictive. We propose to up-weight taxa that are potentially associated with an outcome of interest and down-weight those that are potentially noises using taxon-level association signal strengths. For a binary health outcome, we could apply a two-sample *t*-test to compare abundance levels between the two groups for each taxon and boost the weighted Unifrac and Bray–Curtis distances by the *P*-values of the *t*-tests. To boost unweighted UniFrac and Hamming distances, we could apply the Pearson’s χ^2^ test or Fisher’s exact test to test association between the outcome and a taxon’s presence/absence status. We define taxon-level weights $a_{r}$ as normalized $-{\;\text{log}\;}_{10}\left(p\right)$, where *p* is the *P*-value of the association test. We propose the boosted version of the four distance metrics for microbiome data as follows:

$$\begin{array}{c} d_{w}^{\mathrm{boosted}}(i,j)=\frac{\sum_{r=1}^{R}b_{r}a_{r}\left|p_{ir}-p_{jr}\right|}{\sum_{r=1}^{R}b_{r}a_{r}\left(p_{ir}+p_{jr}\right)},\\ d_{un}^{\mathrm{boosted}}(i,j)=\frac{\sum_{r=1}^{R}b_{r}a_{r}\left|I\left(p_{ir}>0\right)-I\left(p_{jr}>0\right)\right|}{\sum_{r=1}^{R}b_{r}a_{r}I\left(p_{ir}+p_{jr}>0\right)},\\ d_{BC}^{\mathrm{boosted}}(i,j)=\frac{\sum_{r=1}^{q}a_{r}\left|p_{ir}-p_{jr}\right|}{2},\\ d_{H}^{\mathrm{boosted}}(i,j)=\sum_{r=1}^{q}a_{r}\left|I\left(p_{ir}>0\right)-I\left(p_{jr}>0\right)\right|. \end{array}$$

#### 2.1.3 From distance to kernel

The relationship between taxa and the outcome is usually unknown. Thus, we use Gaussian kernel $K\left(i,j\right)=\text{exp}\;\left(-\frac{d{\left(i,j\right)}^{2}}{2\sigma^{2}}\right)$ (Wang *et al.* 2020), which is a universal kernel (Micchelli *et al.* 2006) and can approximate a large class of functions. Here d(i,j) is the distance between samples *i* and *j*, and σ is a parameter that is set as the mean of all pairwise distances among training samples. Note that K(i,j) captures the similarity between samples *i* and *j*.

If we want to use *L* covariates together with microbiome to predict health outcomes, we can calculate e.g. the Euclidian distance between samples *i* and *j* in terms of a covariate, and similarly use Gaussian kernel $K\left(i,j\right)=\text{exp}\;\left(-\frac{d{\left(i,j\right)}^{2}}{2\sigma^{2}}\right)$ or other kernel forms to capture appropriate relationships between covariates and the outcome through *L* kernel matrices $K_{1}^{⋆},\ldots ,K_{L}^{⋆}$. To simplify the notation, we denote kernels for the four boosted distance metrics for microbiome data as $K_{1},\ldots ,K_{4}$ and kernels for *L* covariates as $K_{5},\ldots ,K_{L+4}$.

### 2.2 The proposed multi-kernel model: MK-BMC

To predict a binary outcome utilizing multiple forms of microbiome–outcome associations, we propose the following model that uses the weighted sum of multiple kernels transformed from the proposed boosted distance metrics and distances for covariates:

(1) $$\begin{array}{c} \underset{w}{\text{min}}\{-\sum_{i,j}\sum_{l=1}^{L+4}w_{l}K_{l}(i,j)CC(i,j)+\rho \sum_{l}w_{l}\;\text{log}\;w_{l}\},\\ \;\text{subject to}\;\sum_{l=1}^{L+4}w_{l}=1,w_{l}\geq 0, \end{array}$$

where $w_{l}$ is the weight of kernel *l* and ρ≥0 is a tuning parameter. With *N* training samples, *CC* is a N×N matrix of case–control status:

$$\text{CC}(i,j)=\{\begin{array}{c} 0,\;i=j\\ 1,\;y_{i}=y_{j}\\ -1,\;\;\text{otherwise}. \end{array}$$

The intuition behind the first term in the objective function (1) is that similarities should be relatively small between groups and large within groups. The second term is an entropy loss that encourages equal contributions of multiple kernels. As ρ increases, kernel weights tend to be close to each other. In practice, we set the maximum ρ as the value that achieves max entropy $H(w_{l})=-\sum_{l}w_{l}\;\text{log}\;w_{l}=\log(L+4)$ when $w_{l}=\frac{1}{L+4}$. We tune ρ by considering possible values $\rho \in \{0,\rho_{\max}^{\frac{1}{10}},\ldots ,\rho_{\max}^{\frac{9}{10}},\rho_{\max}\}$ and select the optimal one through 5-fold cross-validations based on the AUC in training samples.

#### 2.2.1 Optimization procedure

Optimizing the objective function (1) is a simple linear programming problem. If the tuning parameter ρ is zero, there only exists one kernel. If ρ>0, we define the (generalized) Lagrangian function with parameters δ>0, and $\sigma_{l}>0,l=1,\ldots ,L+4$ as

$$\begin{array}{c} L(w)=-\sum_{l=1}^{L+4}w_{l}\sum_{i,j}K_{l}(i,j)CC(i,j)+\rho \sum_{l}w_{l}\;\text{log}\;w_{l}\\ -\delta (w^{T}l-1)-\sigma^{T}w. \end{array}$$

By setting $\frac{\partial L(w)}{\partial w_{l}}=0$, it is easy to see that

$$w_{l}=\frac{\;\text{exp}\;\left(\frac{\sum_{i,j}K_{l}(i,j)CC(i,j)}{\rho }\right)}{\sum_{l=1}^{L+4}\;\text{exp}\;\left(\frac{\sum_{i,j}K_{l}(i,j)CC(i,j)}{\rho }\right)}.$$

### 2.3 Building a prediction tool

With estimated kernel weights $w_{l}$, we calculate similarities between samples *i* and *j* as $\sum_{l}w_{l}K_{l}(i,j)$. For sample *i* in a training set with *N* samples, we assign a similarity t-score $t_{i}$ as the two-sample t-statistic comparing its similarities with the remaining cases and controls. With $t_{i}$ and their group label $y_{i},i=1,\ldots ,N$, we fit a simple logistic model $\text{logit}(P\left(y_{i}=1\right))=\beta_{0}+\beta_{1}t_{i}$, which serves as the classifier to predict testing samples’ case–control status.

To predict the case–control status of a testing sample *j*, we compute its similarity with training cases and with training controls separately as $\sum_{l}w_{l}K_{l}(j,i)y_{i}$ and $\sum_{l}w_{l}K_{l}(j,i)(1-y_{i}),i=1\cdots ,N$. We then assign testing sample *j* a t-score $t_{j}$ as the t-statistic comparing these two sets. With $t_{j}$, we can easily calculate the probability of testing sample *j* being a case using the fitted logistic regression classifier.

## 3 Results

### 3.1 Simulation studies

We performed simulation studies to evaluate the prediction performance of the proposed MK-BMC method and that of several competing methods including Random Forest (RF), PAAM-RF, an extended version of RF incorporating the phylogenetic tree structure (Wassan *et al.* 2018), and MDeep, a recently developed deep learning method (Wang *et al.* 2021). MDeep orders OTUs based on a hierarchical clustering analysis using pairwise patristic distances within the phylogenetic tree. The ordered OTUs are subsequently utilized as inputs for a convolutional neural network, enabling predictions that leverage both the phylogenetic tree and OTU abundance levels. We also compared our method with models with single distance metrics or their boosted versions. The single kernel models are denoted as ${\text{SK}}_{BC}$, ${\text{SK}}_{w}$, ${\text{SK}}_{un}$, and ${\text{SK}}_{H}$ representing Bray–Curtis kernel, weighted Unifrac kernel, unweighted Unifrac kernel, and Hamming kernel, respectively. The corresponding boosted versions are denoted as ${\text{SK}}_{BC}^{b}$, ${\text{SK}}_{w}^{b}$, ${\text{SK}}_{un}^{b}$, and ${\text{SK}}_{H}^{b}$, respectively. For RF and PAAM-RF, we set the number of decision trees as 1,000 and the number of variables to possibly split at each node as the (rounded down) square root of the number of variables. All other parameters follow defaults in the R package “ranger.” For MDeep, we used default parameter values on the authors’ GitHub repository (https://github.com/lichen-lab/MDeep). We generated 1,000 datasets, each has a training set and a testing set of equal size *n*. Within each training and testing set, there are an equal number of cases and controls. We considered different sample sizes n=500,200,100.

As with and without covariates, while influencing the overall prediction performance of all methods, it does not fundamentally alter the relative prediction performance of each method, we only included simulation studies without covariates in the main text but included simulation studies with covariates in the supplementary materials.

#### 3.1.1 Simulation settings

Following Chen *et al.* (2012), we simulated microbiome data mimicking a real upper respiratory tract microbiome data (Charlson *et al.* 2010) consisting of 856 OTUs after discarding singletons. Specifically, for sample *i*, the total count $N_{i}$ of 856 OTUs was generated from a negative binomial distribution with mean 1,000 and size 25. Given $N_{i}$, to model the over-dispersion of OTU counts, 856 OTU counts were generated from a Dirichlet-multinomial distribution with proportions $\pi_{1},\ldots ,\pi_{856}$ and an over-dispersion parameter θ, all of which were estimated from the original upper respiratory tract microbiome data and extracted from the R package “MiSPU.” We then transformed OTU counts into OTU abundance levels by dividing total OTU counts of each sample.

To simulate case–control status, we considered three scenarios. Under simulation scenario I, a set of OTUs that are close to each other on the phylogenetic tree were selected as signal OTUs that are associated with the case–control status and thus are referred to as phylogenetically related. Under simulation scenario II, signal OTUs are a set of OTUs that are far away on the phylogenetic tree and thus are referred to as phylogenetically unrelated. Under simulation scenario III, signal OTUs are a mixture of phylogenetically related and unrelated OTUs. Within each scenario, we considered settings when different OTU abundance levels or OTU presence/absence status are associated with the case–control status.

##### 3.1.1.1 Simulation scenario I: signal OTUs are phylogenetically related

To simulate the case–control status $y_{i}$ of sample *i*, we considered two models: Model A uses relative abundances of signal OTUs and Model B uses presence/absence information of signal OTUs:

$$\begin{array}{c} \mathrm{Model}\;A:\;\text{logit}\left\{E\left(y_{i}|p_{i}\right)\right\}=\beta \cdot \mathrm{scale}\left(\sum_{\ell \in G}p_{i\ell }\right),\\ \mathrm{Model}\;B:\;\text{logit}\left\{E\left(y_{i}|p_{i}\right)\right\}=\beta \cdot \mathrm{scale}\left(\sum_{\ell \in G}I\left(p_{i\ell }\right)\right), \end{array}$$

where *G* is the set of signal OTUs, “scale (⋅)” standardizes variables with a mean of 0 and a standard deviation of 1, and I(⋅) is an indicator function. We set all signal OTUs to have the same effect size β for simplicity and considered β=2 or 3.

Under simulation scenario I, to select a set of signal OTUs *G* that are close to each other on the phylogenetic tree, we first partitioned 856 OTU into 20 clusters by partitioning around medoids based on the cophenetic distance matrix using branch lengths on the phylogenetic tree. Numbers of OTUs and total abundance levels of these 20 clusters vary. For Model A, when abundance levels of signal OTUs are related to a binary outcome, we selected the 2nd and 6th most abundant clusters, with 57 and 53 OTUs and total abundance levels 10.39% and 4.91%, respectively, as signal OTUs *G*s. For Model B, when a binary outcome is associated with presence/absence information of signal OTUs, we selected the 8th and 17th most abundant clusters, with 29 and 25 OTUs and total abundance levels 4.59% and 1.43%, respectively, whose average relative abundance per OTU is similar to that of the two clusters used in Model A.

##### 3.1.1.2 Simulation scenario II: signal OTUs are phylogenetically unrelated

Under simulation scenario II, $y_{i}$ was similarly simulated using Models A and B, but signal OTUs are a set of OTUs that are far away from each other on the phylogenetic tree. To do so, we ordered 856 OTUs by their abundance levels and selected a set of signal OTUs *G* as 9 OTUs from nine different clusters with descending abundance levels. For Model A, we selected two sets of nine signal OTUs with total abundance levels 11.14% and 4.77%, respectively. For Model B, we selected another two sets of nine signal OTUs with total abundance levels 10.91% and 2.26%, respectively.

##### 3.1.1.3 Simulation scenario III: Signal OTUs are a mixture of scenarios I and II

Under simulation scenario III with a mixture of phylogenetically related and unrelated signal OTUs, we considered several combinations of signal OTU set:

$$\begin{array}{c} \text{logit}\left\{E\left(y_{i}|p_{i}\right)\right\}=\beta_{1}\cdot \mathrm{scale}(\sum_{\ell \in G_{1}}I(p_{i\ell }))+\beta_{2}\cdot \mathrm{scale}(\sum_{\ell \in G_{2}}I(p_{i\ell }))\\ \text{logit}\left\{E\left(y_{i}|p_{i}\right)\right\}=\beta_{1}\cdot \mathrm{scale}(\sum_{\ell \in G_{1}}I(p_{i\ell }))+\beta_{2}\cdot \mathrm{scale}(\sum_{\ell \in G_{2}}p_{i\ell })\\ \text{logit}\left\{E\left(y_{i}|p_{i}\right)\right\}=\beta_{1}\cdot \mathrm{scale}(\sum_{\ell \in G_{1}}p_{i\ell })+\beta_{2}\cdot \mathrm{scale}(\sum_{\ell \in G_{2}}I(p_{i\ell }))\\ \text{logit}\left\{E\left(y_{i}|p_{i}\right)\right\}=\beta_{1}\cdot \mathrm{scale}(\sum_{\ell \in G_{2}}p_{i\ell })+\beta_{2}\cdot \mathrm{scale}(\sum_{\ell \in G_{2}}I(p_{i\ell })), \end{array}$$

where $G_{1}$ is a set of phylogenetically related OTUs and $G_{2}$ is a set of phylogenetically unrelated OTUs. We set $\beta_{1}=\beta_{2}=2$ or 3.

#### 3.1.2 Simulation results

We evaluated the prediction performance of each method using the area under the ROC curve (AUC), sensitivity, and specificity in testing sets and presented results for n=500,β=2 in the main text. Results for n=500,β=3, and n=200,β=2, and n=100,β=2 are shown in the supplementary materials.

**Figure 1.**
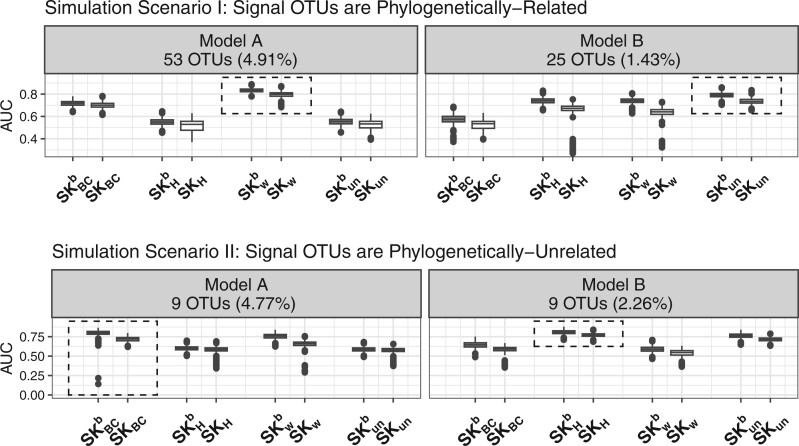
Box plots of AUCs from 1,000 testing sets for the eight single kernel models.

We first investigated if the proposed boosted distance metrics improve prediction performance over the original ones. We compared the prediction performance of two single kernel models with two kernels transformed from either boosted or original distance metrics. Figure 1 displays box plots of AUCs of four pairs of single kernel models from 1,000 simulations for four simulation settings. Single kernel models with kernels that reflect the true microbiome–outcome relationships are in boxes. Complete simulation results of all simulation settings are shown in Figure S1 in the supplementary materials. We observed improved prediction performance of boosted single kernel models over un-boosted versions consistently across almost all simulation settings considered. Models with kernels that reflect the true microbiome–outcome relationships usually benefit the most. This suggests that the proposed boosted distance metrics that up-weight taxa that are potentially associated with the outcome of interest and down-weight taxa that are potentially noises help overall predictions.

We then investigated the prediction performance of the proposed MK-BMC method. Table 1 displays mean AUCs and 0.025 and 0.975 quantiles across 1,000 testing sets for the proposed MK-BMC method and competing methods with the best model in bold. Here “oracle” AUC is calculated from $E(y_{i}|p_{i})$ with true parameter values. Overall, MK-BMC almost always has the best performance or comparable performance to the best competing model when different competing methods perform the best under different simulation settings.

**Table 1.** AUC means and 0.025 and 0.975 quantiles (in parentheses) in testing sets over 1,000 simulations for data generated with β=2.

Simulation scenario I: signal OTUs are phylogenetically related				
Model (association)	A (abundance)	A (abundance)	B (presence/absence)	B (presence/absence)
OTU proportion (number)	10.38% (57)	4.91% (53)	4.59% (29)	1.43% (25)

Oracle	0.858 (0.825, 0.886)	0.849 (0.815, 0.881)	0.849 (0.819, 0.878)	0.851 (0.820, 0.880)
Mdeep	0.797 (0.752, 0.839)	0.777 (0.733, 0.820)	0.542 (0.486, 0.592)	0.561 (0.497, 0.624)
RF	0.747 (0.703, 0.792)	0.714 (0.670, 0.761)	0.701 (0.647, 0.751)	0.709 (0.658, 0.763)
PAAM-RF	0.825 (0.787, 0.863)	0.818 (0.783, 0.856)	0.696 (0.638, 0.746)	0.752 (0.709, 0.794)
${\text{SK}}_{BC}^{b}$	0.755 (0.716, 0.797)	0.717 (0.675, 0.762)	0.581 (0.513, 0.649)	0.575 (0.504, 0.647)
${\text{SK}}_{H}^{b}$	0.532 (0.482, 0.585)	0.550 (0.491, 0.604)	0.735 (0.688, 0.777)	0.740 (0.692, 0.787)
${\text{SK}}_{w}^{b}$	**0.834 (0.799, 0.869)**	**0.832 (0.796, 0.866)**	0.632 (0.554, 0.702)	0.740 (0.695, 0.784)
${\text{SK}}_{un}^{b}$	0.537 (0.485, 0.590)	0.554 (0.501, 0.609)	**0.754 (0.708, 0.795)**	**0.791 (0.745, 0.833)**

MK-BMC	**0.834 (0.798, 0.869)**	0.824 (0.784, 0.861)	0.751 (0.707, 0.792)	0.786 (0.745, 0.830)

Simulation scenario II: signal OTUs are phylogenetically unrelated				
Model (Association)	A (Abundance)	A (Abundance)	B (Presence/Absence)	B (Presence/Absence)
OTU proportion (number)	11.41% (9)	4.77% (9)	10.91% (9)	2.26% (9)

Oracle	0.859 (0.827, 0.889)	0.848 (0.814, 0.882)	0.851 (0.822, 0.880)	0.855 (0.824, 0.885)
Mdeep	0.780 (0.734, 0.823)	0.746 (0.693, 0.794)	0.568 (0.508, 0.624)	0.579 (0.526, 0.636)
RF	0.762 (0.715, 0.806)	0.756 (0.707, 0.799)	0.766 (0.718, 0.810)	0.762 (0.716, 0.808)
PAAM-RF	0.761 (0.712, 0.808)	0.743 (0.686, 0.794)	0.747 (0.697, 0.795)	0.700 (0.642, 0.754)

${\text{SK}}_{BC}^{b}$	**0.804 (0.753, 0.847)**	**0.798 (0.741, 0.848)**	0.632 (0.558, 0.694)	0.643 (0.569, 0.710)
${\text{SK}}_{H}^{b}$	0.535 (0.485, 0.592)	0.601 (0.540, 0.661)	**0.792 (0.744, 0.839)**	**0.808 (0.749, 0.855)**
${\text{SK}}_{w}^{b}$	0.754 (0.691, 0.810)	0.755 (0.680, 0.815)	0.587 (0.515, 0.656)	0.588 (0.514, 0.658)
${\text{SK}}_{un}^{b}$	0.523 (0.476, 0.576)	0.586 (0.529, 0.643)	0.760 (0.712, 0.806)	0.763 (0.703, 0.816)

MK-BMC	0.779 (0.726, 0.825)	0.783 (0.720, 0.838)	0.788 (0.741, 0.837)	0.804 (0.746, 0.854)

Simulation scenario III: signal OTUs are a mixture of scenarios I and II				
Association	$P_{\mathrm{phy}}+P_{\mathrm{unphy}}$	$P_{\mathrm{phy}}+A_{\mathrm{unphy}}$	$A_{\mathrm{phy}}+P_{\mathrm{unphy}}$	$A_{\mathrm{unphy}}+P_{\mathrm{unphy}}$
OTU proportion (number)	4.59%+10.91% (29 + 9)	1.43%+11.41% (25 + 9)	10.38%+2.26% (57 + 9)	11.41%+2.26% (9 + 9)

Oracle	0.906 (0.881, 0.929)	0.910 (0.887, 0.934)	0.910 (0.883, 0.933)	0.910 (0.886, 0.934)
Mdeep	0.559 (0.502, 0.611)	0.719 (0.666, 0.768)	0.725 (0.681, 0.771)	0.724 (0.676, 0.769)
RF	0.743 (0.693, 0.788)	0.750 (0.707, 0.796)	0.773 (0.725, 0.817)	0.777 (0.731, 0.825)
PAAM-RF	0.721 (0.668, 0.770)	0.769 (0.729, 0.811)	0.771 (0.726, 0.813)	0.731 (0.678, 0.783)

${\text{SK}}_{BC}^{b}$	0.605 (0.534, 0.667)	0.723 (0.671, 0.776)	0.700 (0.653, 0.746)	0.730 (0.675, 0.783)
${\text{SK}}_{H}^{b}$	0.775 (0.719, 0.826)	0.671 (0.614, 0.721)	0.728 (0.675, 0.781)	0.727 (0.675, 0.776)
${\text{SK}}_{w}^{b}$	0.604 (0.528, 0.669)	0.741 (0.689, 0.791)	0.753 (0.712, 0.793)	0.686 (0.622, 0.745)
${\text{SK}}_{un}^{b}$	0.763 (0.707, 0.817)	0.711 (0.662, 0.760)	0.692 (0.634, 0.748)	0.689 (0.635, 0.743)

MK-BMC	**0.782 (0.724, 0.833)**	**0.790 (0.743, 0.834)**	**0.788 (0.741, 0.833)**	**0.796 (0.739, 0.852)**

The best models are indicated in bold.

More specifically, in simulation scenarios I and II, single kernel models with kernels reflecting true microbiome–outcome relationships always perform the best, while the proposed MK-BMC achieves comparable performance to that of the best single kernel model. In simulation scenario III, when signal OTUs are a mixture of phylogenetically related and unrelated, presence/absence and abundant OTUs, i.e., under scenarios that MK-BMC was designed for, MK-BMC outperforms all competing methods.

**Figure 2.**
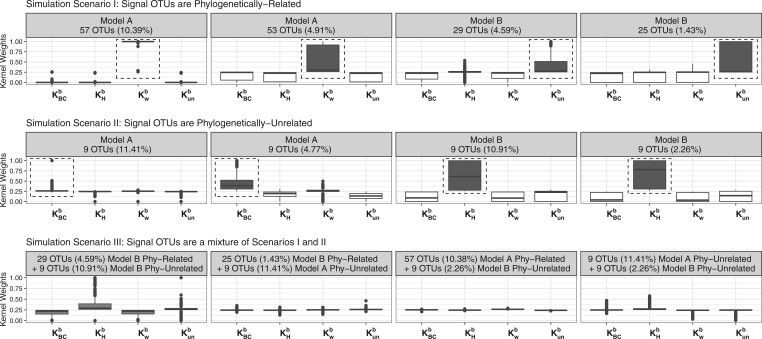
Box plots of kernel weights of the four kernels in the proposed MK-BMC method from 1,000 training sets.

Moreover, kernel weights give us insights into types of contributing OTUs. Figure 2 displays box plots of kernel weights of MK-BMC. We notice that weights for kernels that reflect the true microbiome–outcome relationships are the largest for all simulation settings in simulation scenarios I and II, while in simulation scenario III, four kernel weights are more similar with kernels representing true microbiome–outcome relationships being slightly larger. For example, under the second setting in simulation scenario III with mixtures of phylogenetically related and unrelated presence/absence (Model B) signal OTUs, kernel $K_{H}^{b}$ has the largest weight followed by kernel $K_{un}^{b}$, while the weights of kernels $K_{BC}^{b}$ and $K_{w}^{b}$ are small. This is promising for real microbiome studies when true associations between microbiome and outcomes are complicated and unknown.

For competing methods, PAAM-RF that uses tree information almost always outperforms RF when signal OTUs are phylogenetically related. When signal OTUs are phylogenetically unrelated, RF performs better than PAAM-RF. The deep learning method MDeep performs worse than PAAM-RF in most simulation settings and does not have much predictability with signal OTUs being presence/absence.

We included sensitivity and specificity results in the Supplementary Tables S2, S3, S5, S6, S8, and S9, where the cutoff for classifying cases/controls for all methods is 0.5. We can see that, across all simulation settings, no single method consistently outperforms others in terms of both sensitivity and specificity. There is a trade-off between them, methods with higher sensitivity than others tend to have lower specificity, and vice versa. Only under simulation settings, when the presence/absence information of abundant phylogenetically related signal OTUs is related to a health outcome, the proposed MK-BMC performs the best in all three metrics, AUCs, sensitivities, and specificities across all methods.

We studied the impact of signal density when signal OTUs are phylogenetically related or unrelated (Supplementary Table S11). To do so, we fixed total abundance levels of all signal OTUs but increased the number of signal OTUs to increase the “signal density.” Thus, the abundance level per signal OTU decreases as signal density increases. As expected, when signal OTUs are phylogenectically unrelated, AUCs of MK-BMC and all competing methods decrease with increasing number of signal OTUs while fixing the total abundance level. However, when signal OTUs are phylogenectically related, AUCs of MK-BMC and several competing methods that use the phylogenetic tree information improve with increasing number of signal OTUs when fixing the total abundance level. This is because with more signal OTUs that are close to each other on the phylogenetic tree, MK-BMC, PAAM, Mdeep, and single kernel methods ${\text{SK}}_{w}$ and ${\text{SK}}_{w}^{b}$ can use more of the phylogenetic tree information and thus have improved prediction performance. See Supplementary Section A5 for more details.

For additional simulation studies with covariates, in general, we observed similar prediction patterns with/without covariates and the prediction performance of all methods improves as the effect size of covariates increases as expected. Moreover, kernel weights of covariates in MK-BMC also increase with increasing effect size of covariates.

### 3.2 Applications to the American Gut Project

We applied MK-BMC and competing methods to the microbiome data from the American Gut Project (AGP, McDonald *et al.* 2018, http://americangut.org; EBI: ERP012803) to predict multiple binary health outcomes. To evaluate the prediction performance, we randomly split samples into equally sized training and testing data 1000 times. We trained MK-BMC and competing methods using training data and evaluated their prediction performance using AUCs in testing data.

AGP was launched in 2012 to better understand the role of microbes in health. AGP participants provided detailed self-reported metadata. Microbiome samples were collected from different body habitats including fecal, oral, skin, and other body sites. We downloaded the latest version of the processed OTU count table (similarity level 97%), which includes 19 524 samples and 36 405 OTUs from ftp://ftp.microbio.me/AmericanGut/ag-2017-12-04/03-otus.zip/100nt/gg-13_8-97-percent/otu_table.biom. We also downloaded health-related information from https://qiita.ucsd.edu/study/description/10317. We considered 4749 samples out of the 19 524 samples whose “country” was “USA” and “country residence” was “United States”. We further removed samples with total OTU counts less than 1250 (McDonald *et al.* 2018), thus yielding 4,620 samples. We focused on gut samples and considered three binary outcomes for predictions: thyroid status, obesity status, and inflammatory bowel disease (IBD) status.

We incorporated covariate age to enhance prediction performance for each binary outcome. Samples without age information were excluded. To make prediction results with and without age comparable for each outcome, we used the same set of samples to conduct predictions with microbiome only, age only, and microbiome + age. For RF and PAAM-RF, we included age during every node split in addition to the randomly selected subset of OTUs to make sure age is always in the model. All parameters for RF and PAAM-RF were set the same as in simulation studies. Note that the deep learning method MDeep was not implemented to handle covariates, and thus it was not included as a competing method here. Detailed information on the three outcomes, data processing steps, and prediction results using microbiome only with larger sample sizes without considering missing age are included in the supplementary materials.

Table 2 summarizes AUC means and 0.025 and 0.975 quantiles in testing sets across 1,000 50/50 random splits for the three outcomes with the best models in bold. MK-BMC consistently performs the best or as well as the best model across all methods for the three outcomes. As shown in Supplementary Figure S8 with box plots of AUCs of eight single kernel models, we noticed that single kernel models using boosted distance metrics have better and more stable performance across different random splits than that of single kernel models using original distance metrics in general.

**Table 2.** Summary of the three outcomes together with AUC means and 0.025 and 0.975 quantiles (in parentheses) in testing sets across 1000 50/50 random splits.

	Thyroid	Obesity	IBD
	Gut	Gut	Gut
#samples	2,941	1,930	2,913
#cases (%)	355 (12.1%)	348 (18.0%)	136 (4.7%)
#OTUs	13,115	11,841	13,028

Age only			

Regression	0.633 (0.606, 0.661)	0.649 (0.620, 0.676)	0.516 (0.413, 0.565)
${\text{SK}}_{\mathrm{cov}}$	0.636 (0.606, 0.663)	0.651 (0.620, 0.682)	0.506 (0.418, 0.573)

Microbiome only			

RF	0.554 (0.519, 0.587)	0.597 (0.558, 0.637)	0.670 (0.622, 0.717)
PAAM-RF	0.572 (0.538, 0.606)	0.649 (0.612, 0.685)	0.677 (0.630, 0.723)

${\text{SK}}_{BC}^{b}$	0.597 (0.562, 0.631)	0.613 (0.562, 0.659)	0.656 (0.588, 0.721)
${\text{SK}}_{H}^{b}$	0.607 (0.571, 0.644)	0.651 (0.607, 0.695)	0.681 (0.630, 0.732)
${\text{SK}}_{w}^{b}$	0.569 (0.526, 0.614)	0.605 (0.550, 0.653)	0.634 (0.558, 0.697)
${\text{SK}}_{un}^{b}$	0.608 (0.567, 0.647)	0.646 (0.603, 0.692)	0.686 (0.632, 0.739)

MK-BMC	0.607 (0.566, 0.648)	0.650 (0.605, 0.694)	**0.688 (0.626, 0.742)**

Microbiome + age			

RF	0.614 (0.582, 0.644)	0.691 (0.654, 0.724)	NA^a^
PAAM-RF	0.614 (0.580, 0.647)	**0.713 (0.674, 0.748)**	NA

MK-BMC	**0.647 (0.594, 0.686)**	0.700 (0.633, 0.755)	NA

a Age is not predictive for IBD.

The best models are indicated in bold.

**Figure 3.**
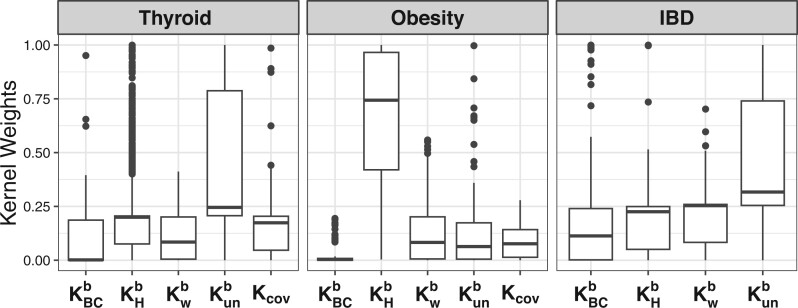
Box plots of kernel weights of the kernels in the proposed MK-BMC method from testing sets across 1,000 50/50 random splits.

When predicting thyroid status based solely on microbiome information, the proposed MK-BMC has the best AUC across all methods, which is as good as that of ${\text{SK}}_{H}^{b}$ and ${\text{SK}}_{un}^{b}$. Moreover, ${\text{SK}}_{BC}^{b}$ also has similar AUCs, which indicates that a mixture of abundant phylogenetic-unrelated taxa and rare phylogenetic-related taxa is predictive of thyroid status. This observation is also confirmed by the fact that PAAM-RF slightly outperforms RF. Age is a stronger predictor than microbiome for thyroid status, with a mean AUC around 0.63. When considering both microbiome and age as predictors, MK-BMC performs the best with a mean AUC of 0.647. However, for RF and PAAM-RF, predictions using both microbiome and age have lower performance than that of using age only, although better than microbiome only. This is because for RF and PAAM-RF, with large number of OTUs whose effects are small, the effect of age can be easily buried. On the other hand, MK-BMC treats age and microbiome as distinct kernels thus can effectively capture age signal even in the presence of a large number of OTUs. We further investigated estimated kernel weights in MK-BMC (Fig. 3) and noticed that kernel $K_{un}^{b}$ has the largest weight, while $K_{H}^{b}$, $K_{\mathrm{cov}}$, $K_{BC}^{b}$, and $K_{w}^{b}$ have similar weights, which is consistent with the findings from single kernel models, suggesting that a mixture of abundant phylogenetic-unrelated taxa and rare phylogenetic-related taxa together with age is predictive of thyroid status.

When predicting obesity status based solely on microbiome information, the proposed MK-BMC has the best AUC across all methods, which is as good as that of ${\text{SK}}_{H}^{b}$ and ${\text{SK}}_{un}^{b}$. This indicates that a mixture of rare microbiome profiles that are phylogenetically related and unrelated is predictive of obesity. Age itself is also predictive of obesity with a mean AUC of about 0.65. When age is incorporated together with microbiome, MK-BMC has a mean AUC of 0.700, which is close to that of the best method PAAM-RF with a mean AUC of 0.713. The performance of MK-BMC, RF, and PAAM-RF all improved adding age. This is because the effect size of both microbiome and age is strong. In terms of estimated kernel weights, kernel $K_{H}^{b}$ has the largest weight and $K_{BC}^{b}$ barely has any weight, while $K_{w}^{b}$, $K_{un}^{b}$, and $K_{\mathrm{cov}}$ have similar weights, again suggesting that a mixture of rare microbiome profiles that are phylogenetically related and unrelated together with age is predictive of obesity status.

For IBD status, age is not predictive with a mean AUC around 0.51. Thus, we only fit two models, i.e. age only and microbiome only. With microbiome only, MK-BMC outperforms all other methods with a mean AUC of 0.688. PAAM-RF performs slightly better than RF. Further investigation of estimated kernel weights in MK-BMC shows that all four kernels have relatively similar weights, while weights of kernels $K_{W}^{b}$ and $K_{un}^{b}$ are slightly larger. This indicates that some taxa either rare or abundant that are phylogenetically related are predictive of IBD.

## 4. Discussion

In this paper, we developed MK-BMC, a multi-kernel model with boosted distance metrics for microbiome data for classification. With several widely used distance metrics for microbiome data including weighted and unweighted UniFrac distances and Bray and Curtis distance, the proposed boosted distance metrics up-weight taxa that are potentially associated with an outcome of interest and down-weight taxa that are potentially noises. MK-BMC then uses multiple kernels transformed from the proposed boosted distance metrics to consider multiple forms of microbiome–outcome associations and thus can use multiple prediction signals to improve overall prediction performance. The learned kernel weights by MK-BMC give insights into contributions of different types of taxa on an overall prediction.

In simulation studies covering a wide range of scenarios, we demonstrated the advantages of the proposed boosted distance metrics that use taxon-level signal strengths for overall predictions over original ones. Similar ideas that up-weight potential signal features and down-weight potential noise features in distance-based methods have been used in other types of omics data (Ruan *et al.* 2019, Wang *et al.* 2019) for disease subtyping or for disease signal identifications. We also showed the much-improved prediction performance of MK-BMC over competing methods in almost all simulation scenarios considered. We observed that (i) when signal OTUs are a mixture of different types of OTUs, e.g. either phylogenetically related or unrelated, etc., i.e., scenarios MK-BMC was designed for, MK-BMC always performs the best; and (ii) when signal OTUs are single type of OTUs, MK-BMC performs almost always as well as the single kernel model with the kernel that reflects the true microbiome–outcome association.

We applied MK-BMC and competing methods to predict binary thyroid, obesity, and IBD status using gut microbiome data from the AGP while incorporating age as a covariate. MK-BMC consistently performs the best or as well as the best model across all methods for the three outcomes. Moreover, for outcomes where age and microbiome are both predictive, MK-BMC consistently improves when incorporating age, while prediction performance of RF and PAAM-RF with both age and microbiome may sometimes be worse than that with age only depending on how strongly age and OTUs are predictive. Furthermore, kernel weights from MK-BMC provide information on the contributions of different types of microbiome profiles in predicting these outcomes.

To boost individual taxon in calculating distance metrics for microbiome data, both taxon-level *P*-values and effect sizes are potential choices. We compared prediction performance of these two types of boosting weights with different sample sizes. We observed that prediction results with *P*-values being boosting weights are more stable than that with effect sizes being boosting weights as sample sizes decrease. This is because *P*-value calculations consider variations in effect size estimates and thus are less affected by sample sizes. Nevertheless, it is noteworthy that, due to the boosting process, kernel weights may not remain stable when the sample size is small. Despite this, MK-BMC demonstrates robust performance across various sample sizes.

While we used Gaussian kernel for covariates, MK-BMC has the flexibility to incorporate linear or other kernel forms to capture appropriate covariate–outcome relationships. While we only considered binary outcomes, with continuous health outcomes, we could use kernel regressions based on the proposed boosted distance metrics for microbiome data.

## Supplementary Material

btad757_Supplementary_Data

## Data Availability

Source code together with a sample input dataset is available at https://github.com/HXu06/MK-BMC.
