# Supplementary material for: MK-BMC: a Multi-Kernel framework with Boosted distance metrics for Microbiome data for Classification
Source: Bioinformatics. 2024 Jan 10;40(1):btad757. doi: 10.1093/bioinformatics/btad757 (PMC10789312; doi:10.1093/bioinformatics/btad757)
Supplement: btad757_Supplementary_Data [file btad757_supplementary_data.pdf]

Supporting Information for "MK-BMC: A Multi-Kernel framework with Boosted distance metrics for Microbiome data for Classification"

Huang Xu[1],Tian Wang[2], Yuqi Miao[2], Min Qian[2], Yaning Yang[1], and Shuang Wang[2]

[1]Department of Statistics and Finance, University of Science and Technology of China, Hefei, Anhui, P. R. China

[2]Department of Biostatistics, Mailman School of Public Health, Columbia University, New York, New York, U.S.A.

A. Additional simulation studies

A.1 Simulation studies with covariates

We conducted additional simulation studies to investigate the prediction performance of the proposed MK-BMC with covariates. We fixed the training sample size as 250 cases and 250 controls and considered one binary covariate with a binomial distribution with a probability of 0.5. The case/control status was determined based on the model:

$$\text{Model A:} \quad \operatorname{logit}\left\{E\left(y_i \mid \mathbf{p}_i, X_i\right)\right\} = \beta \cdot scale\left(\sum_{\ell \in G} p_{i\ell}\right) + \beta_{cov} \cdot X_i.$$

We set $\beta = 2$ and varied $\beta_{cov} = 1.5, 2, 2.5$. We considered 2 scenarios of signal OTUs. The first scenario has 57 phylogenetically-related signal OTUs with a total abundance 10.38%. The second scenario has 9 phylogenetically-unrelated signal OTUs with a total abundance 11.41%. As the deep learning method MDeep was not implemented to handle covariates, it was not included as a competing method here. Table S1 displays AUC means and 0.025 and 0.975 quantiles (in parentheses) together with kernel weights in MK-BMC in testing sets over 1,000 simulations. In general, the performance of MK-BMC, RF, and PAAM-RF improves as the effect size of covariates increases as expected. What's more, the kernel weight of the binary covariate in MK-BMC also increases as the effect size increases.

A.2 Additional simulation results of single kernel models with AUCs

Figure S1 displays AUCs of 8 single kernel models of the three simulation scenarios considered where $\beta = 2$ with 250 cases and 250 controls. We boxed single kernel models with kernels that reflect the true microbiome-outcome relationships. We observed improved prediction performance of single kernel models using the proposed boosted distance metrics for microbiome data over that of the single kernel models using the original distance metrics across almost all simulation settings considered. Moreover, models with kernels that reflect the true microbiome-outcome relationships usually benefit the most except when the single kernel models using the original distance metrics already perform well enough. This suggests that the proposed boosted distance metrics that up-weight taxa that are potentially associated with the outcome of interest and down-weight taxa that are potentially noises help overall prediction.

A.3 Additional simulation results with sensitivity and specificity

We also used sensitivity and specificity as evaluation metrics. The cutoff for classifying cases/controls for all methods is 0.5. Table S2 and Table S3 display sensitivities and specificities of the three simulation scenarios considered where $\beta = 2$ with 250 cases and 250 controls. We can see that, across these simulation settings, no single method consistently outperforms others in terms of both sensitivity and specificity. There is a trade-off between sensitivity and specificity, methods with higher sensitivity than others tend to have lower specificity, and vice versa. Only under simulation settings when the presence/absence information of abundant phylogenetically-related signal OTUs is related to a health outcome, the proposed MK-BMC performs the best in all three metrics, AUCs, sensitivities, and specificities across all methods.

A.4 Additional simulation studies with different sample sizes and different effect sizes of signal OTUs

We also conducted simulation studies with different training sample sises n and different effect size β of signal OTUs.

Tables S4, S5 and S6 display AUCs, sensitivities and specificities when $\beta = 2$ with 100 cases and 100 controls. Tables S7, S8 and S9 display AUCs, sensitivities and specificities when $\beta = 2$ with 50 cases and 50 controls. Table S10 displays AUC when $\beta = 3$ with 250 cases and 250 controls.

The overall prediction patterns of MK-BMC and competing methods are similar across different sample sizes and different effect sizes β of signal OTUs. Nevertheless, it's noteworthy that, due to the boosting process, kernel weights may not remain stable when the sample size is small.

A.5 Additional simulation studies to investigate signal density

We conducted additional simulations to study the impact of signal density. Note that, to do so, we fixed total abundance levels of all signal OTUs but increased the number of signal OTUs to increase the "signal density". Thus, the abundance level for individual signal OTUs decreases as signal density increases. We studied the situations when the abundance of signal OTUs that are phylogenetically-related and -unrelated is associated with a binary outcome. We considered sets of signal OTUs that have similar total abundance levels but different numbers of OTUs when fixing sample size as 250 cases and 250 controls. For the phylogenetically-related signal OTUs, we considered a set of 29 OTUs with a total abundance level of 4.59% and a set of 53 OTUs with a total abundance level of 4.91%, respectively. For the phylogenetically-unrelated signal OTUs, we considered a set of 9 OTUs with a total abundance level of 4.77% and a set of 18 OTUs with a total abundance level of 4.84%, respectively. Table S11 displays the AUC means and 0.025 and 0.975 quantiles (in parentheses) in testing sets over 1000 simulations for data generated with different signal densities.

As expected, when signal OTUs are far away from each other on the phylogenetic tree, i.e., phylogenectically-unrelated, AUCs of the proposed MK-BMC and all competing

methods decrease with the increasing total number of signal OTUs while fixing the total abundance level of signals.

However, when signal OTUs are close to each other on the phylogenetic tree, i.e., phylogenectically-related, AUCs of the proposed MK-BMC and several competing methods that use the phylogenetic tree information improve with the increasing number of signal OTUs when fixing the total abundance level of signals. This is because with more signal OTUs that are close to each other on the phylogenetic tree, the proposed MK-BMC method, PAAM, Mdeep and single kernel methods SK_w and SK_w^b can use more of the phylogenetic tree information, thus have improved performance with the increasing number of signal OTUs with a fixed total signal OTU abundance levels.

A.6 Additional simulation studies to investigate ways to boost signals in distances

To boost individual taxon in calculating distance metrics for microbiome data, both taxon-level p-values and effect sizes are potential choices. We define boost weight for taxon r as $a_r = \frac{|\beta_r|}{\sum |\beta_r|}$ with effect size β_r, where β_r is the regression coefficient estimate of taxon r in a logistic regression associating taxon r and a binary outcome. For the weighted Unifrac and Bray-Curtis distances that use the abundance information, the relative abundance of each taxon is used in the logistic regression to estimate β_r. For the unweighted UniFrac and Hamming distances that use the presence/absence information, the presence/absence status of each taxon is used in the logistic regression.

We studied the performances of using boosting weights based on effect size and p-values at different training sample sizes. We considered one simulation scenario when abundant information (total abundance level as 10.38% with 57 signal OTUs) is associated with a binary outcome and signal OTUs are phylogenetically-related. We considered different training sample sizes ranging from 500 to 100 with half cases and half controls. Table S12 displays the AUC means and 0.025 and 0.975 quantiles (in parentheses) in testing sets over 1000 simulations for data generated with abundant phylogenetically-related signal OTUs. We can see that as training sample sizes decrease, all methods have decreased AUCs, but the decrease in MK-BMC when using p-values to boost the signals is smaller than the decrease in MK-BMC when using regression coefficients to boost signals. That is because

p-values that consider variations in estimates, will be more stable than effect size estimates, especially when sample sizes are small.

B. Additional results for real data applications

We predicted three outcomes using microbiome only without considering age, thus there are bigger sample sizes for each of the three outcomes. Figure S8 displays the AUCs of 8 single kernel models predicting 3 binary outcomes in our real data applications. In general, single kernel models using kernels from the proposed boosted distance metrics perform better than single kernel models using the original distance metrics. What's more, single kernel models using kernels from the proposed boosted distance metrics are usually more robust and less likely to reverse cases and controls thus with fewer values smaller than 0.5. However, due to the small proportion of cases, single kernel models using kernels from the proposed boosted distance metrics sometimes perform slightly worse than single kernel models using the original distance metrics.

The results of the 3 binary outcomes in our applications when the covariate "age" is not included nor used for sample filtering are similar to those when age is not included but used for sample filtering (i.e. the microbiome only results in the main text). Table S13 displays AUC means and 0.025 and 0.975 quantiles in testing sets across 1,000 50/50 random splits with the best models in bold when age is not included nor used for sample filtering. Although MDeep is included as a competing method here, MDeep does not perform as well as the other competing methods and the proposed MK-BMC method.

C. Real Data Preprocessing Procedure

C.1 Data Access

We downloaded the latest version of the OTU count table which includes 19,524 samples and 36,405 OTUs from `ftp://ftp.microbio.me/AmericanGut/ag-2017-12-04/03-otus.zip/100nt/gg-13_8-97-percent/otu_table.biom`. Specifically, the V4 region of 16S rRNA gene fragments was sequenced after being amplified with barcoded primers, and then the 16S sequence data were trimmed to 100 nucleotides and processed in QIIME (version: 1.9.1)

[1] to perform closed-reference OTU picking at similarity level 97% against Greengenes (version: 13.8) [4].

The health-related information was downloaded from `https://qiita.ucsd.edu/study/description/10317`. We considered 4,749 samples of the 19,524 whose "country" was "USA" and "country residence" was "United States". The samples were further selected following the criteria outlined in [3] by removing those with total OTU counts less than 1,250 and thus yielding 4,620 samples. Table S14 illustrates the distribution of body habitats from which the 11,910 samples were collected. We mainly focused on the gut samples.

C.2 Selected Outcomes

We selected 3 binary health outcomes that were potentially associated with the human microbiome. For these 3 outcomes, we listed variable names and the corresponding responses in Table S15. We incorporated age as a non-microbiome-related covariate to enhance prediction performance for each outcome. To ensure data completeness, samples with missing age information were filtered out. Table S16 provides details on sample sizes, numbers (percentages) of cases for gut microbiome samples, and the corresponding number of OTUs based on these samples. The data is presented separately for scenarios where age information is utilized for sample filtering and where it is not.

C.3 Phylogenetic Tree Construction

A reference phylogenetic tree for OTUs at 97% similarity is provided in the Greengenes database. The branch length of the 4,892-th edge is missing from this tree, so we set it to 0. The original tree contains 99,322 leaf nodes (i.e., OTUs). With different outcomes having different numbers of samples, we created individual phylogenetic trees for individual outcomes, whose leaf nodes are restricted to OTUs that exist in samples for individual outcomes and microbiome body habitat sites. We used the R package `castor` [2].

D. Supplementary Figures

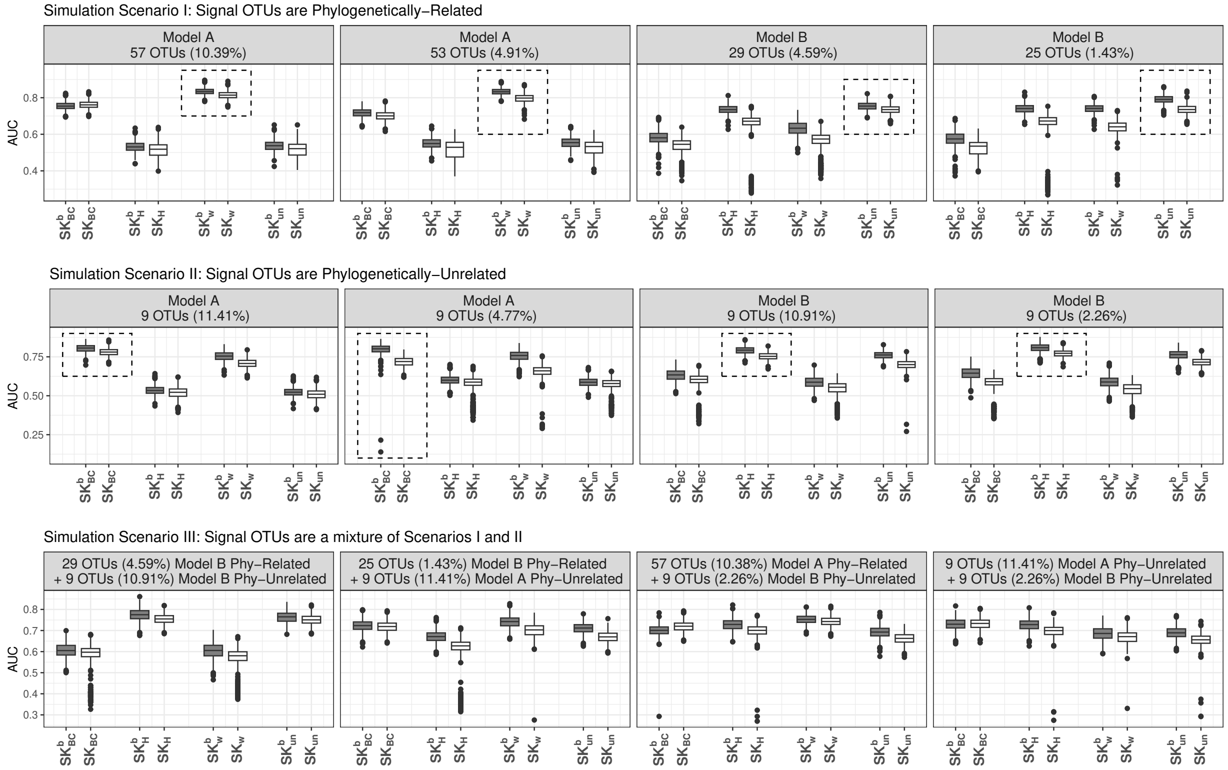

Figure S1: Box plots of AUCs in 1,000 test sets for the 8 single kernel models with $\beta = 2, n = 500$.

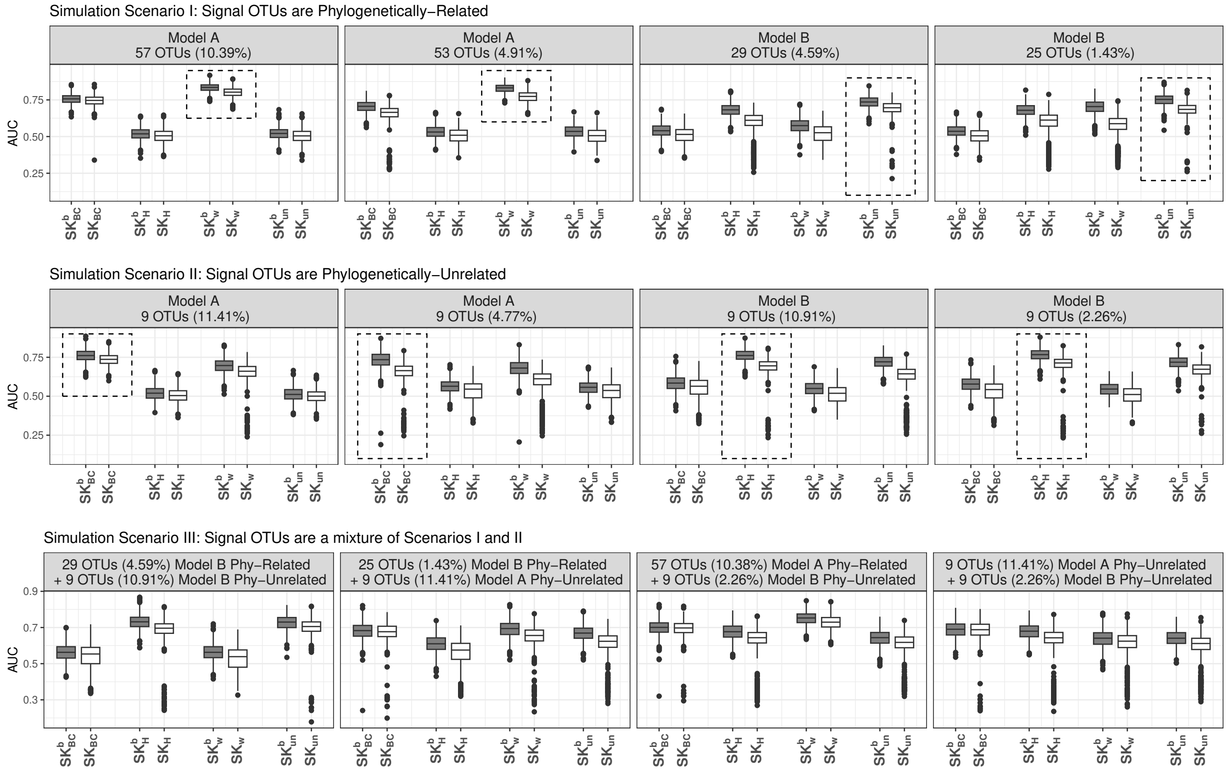

Figure S2: Box plots of AUCs in 1,000 test sets for the 8 single kernel models with $\beta = 2, n = 200$.

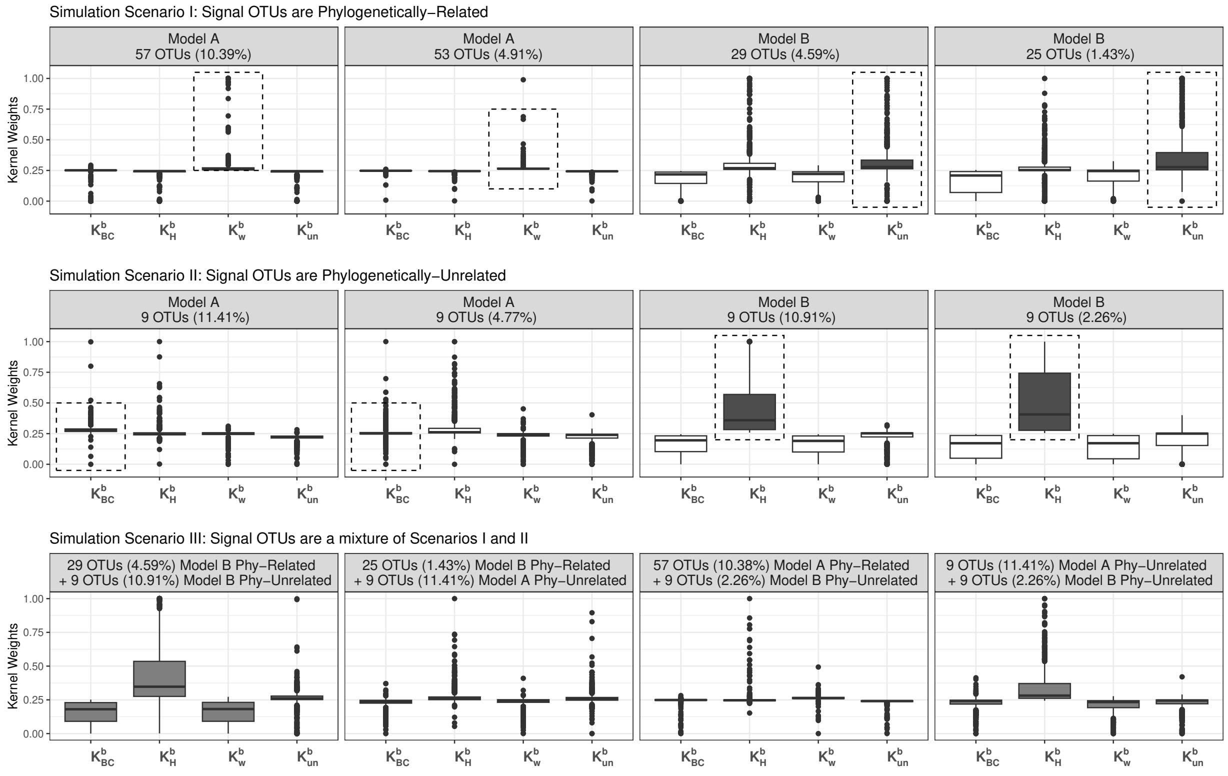

Figure S3: Box plots of kernel weights of the 4 kernels in the proposed MK-BMC method from 1,000 training sets with $\beta = 2, n = 200$.

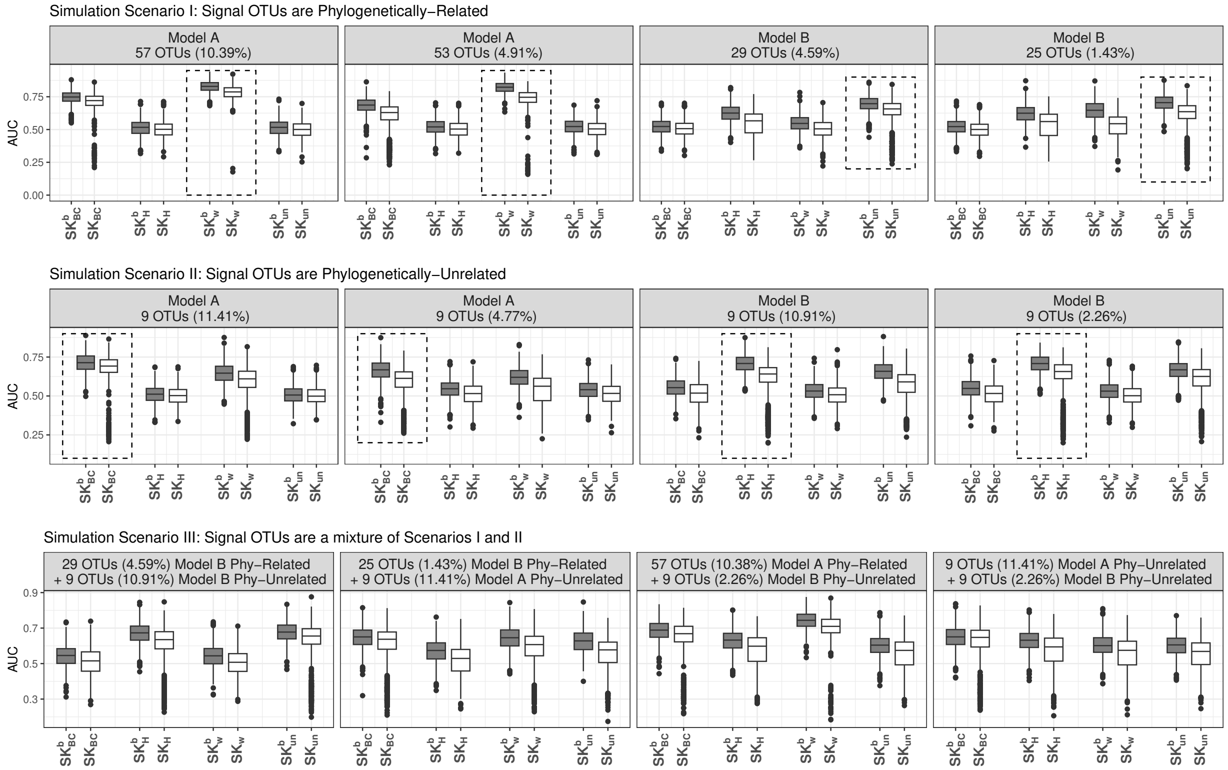

Figure S4: Box plots of AUCs in 1,000 test sets for the 8 single kernel models with $\beta = 2, n = 100$.

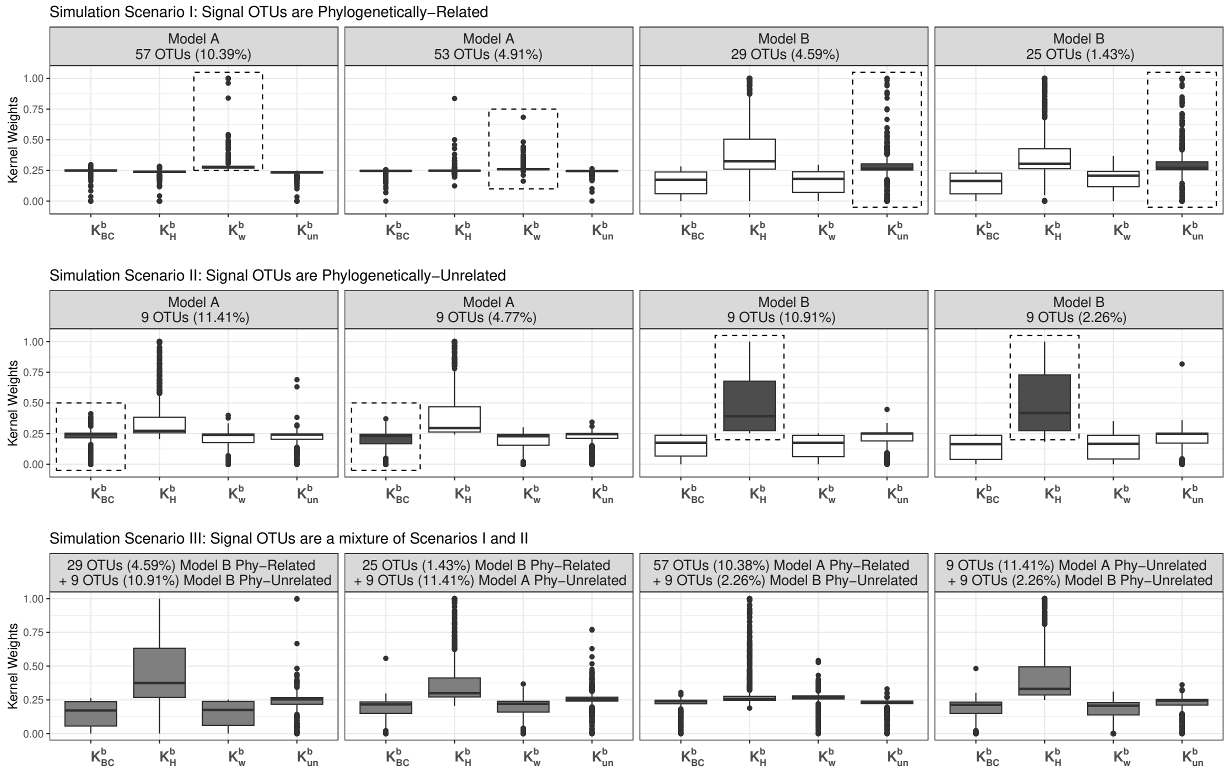

Figure S5: Box plots of kernel weights of the 4 kernels in the proposed MK-BMC method from 1,000 training sets with $\beta = 2, n = 100$.

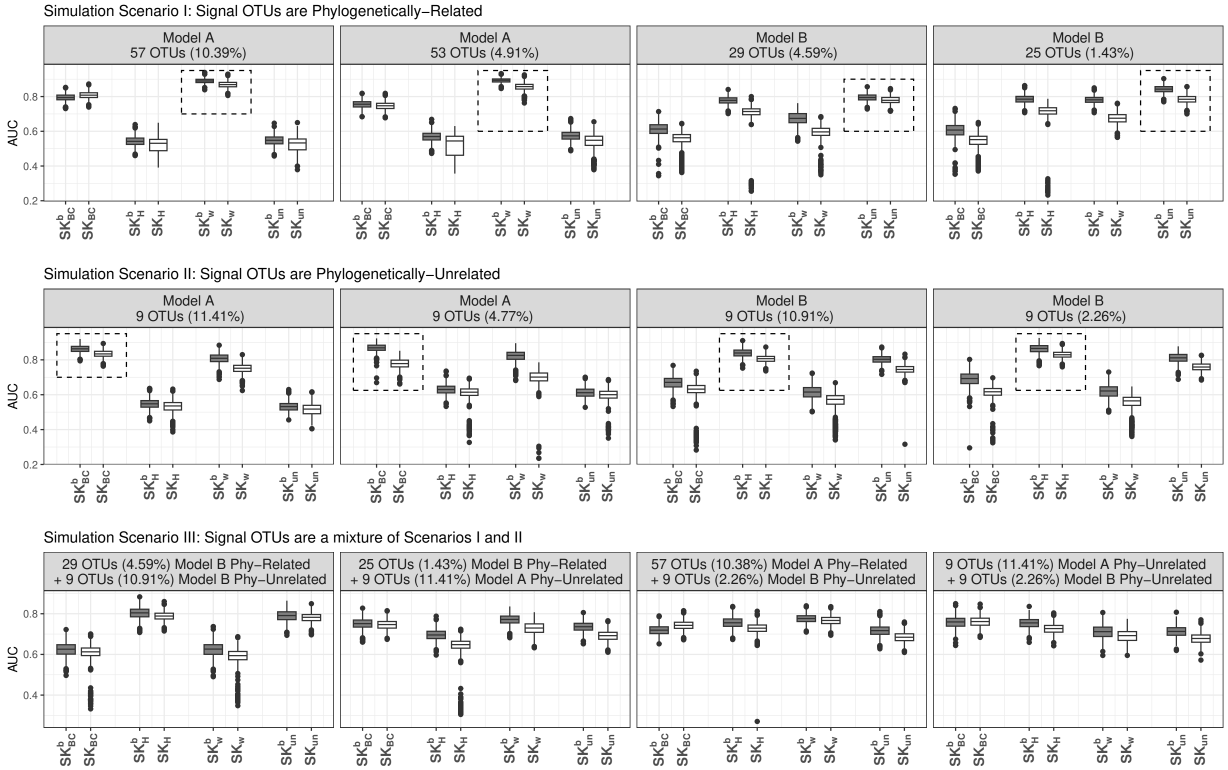

Figure S6: Box plots of AUCs in 1,000 test sets for the 8 single kernel models with $\beta = 3, n = 500$.

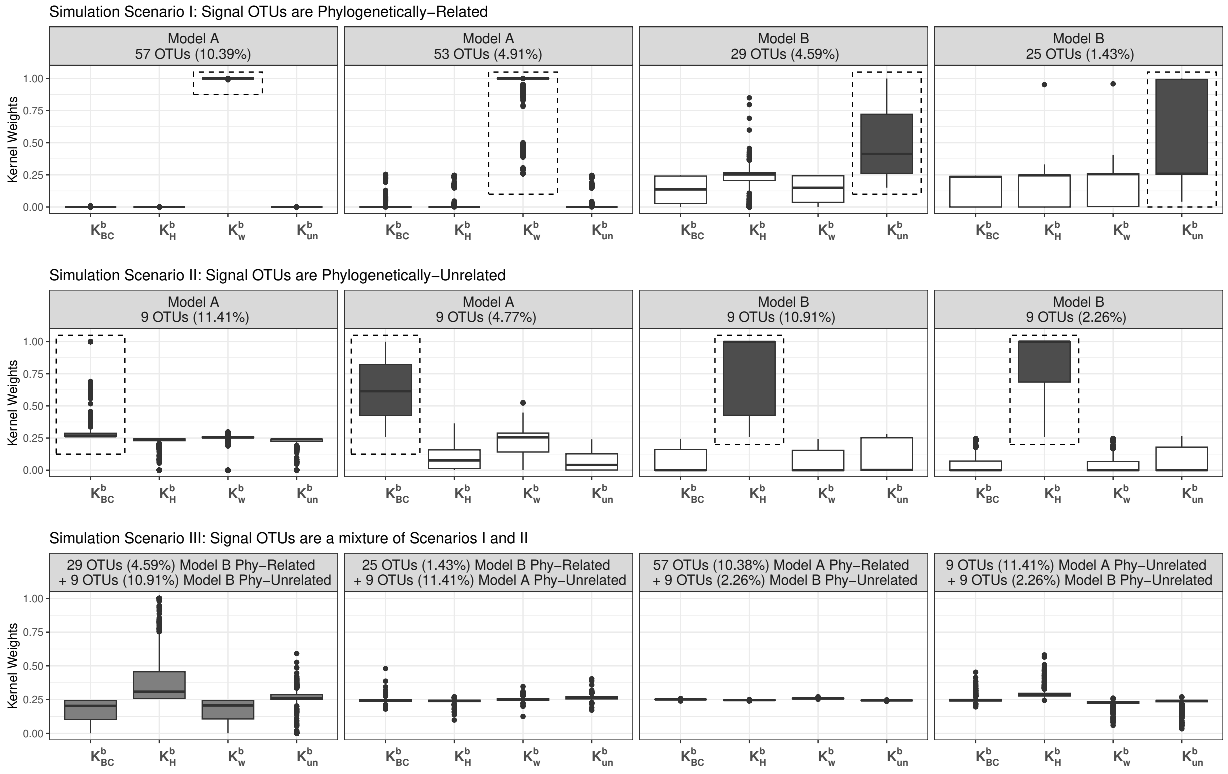

Figure S7: Box plots of kernel weights of the 4 kernels in the proposed MK-BMC method from 1,000 training sets with $\beta = 3, n = 500$.

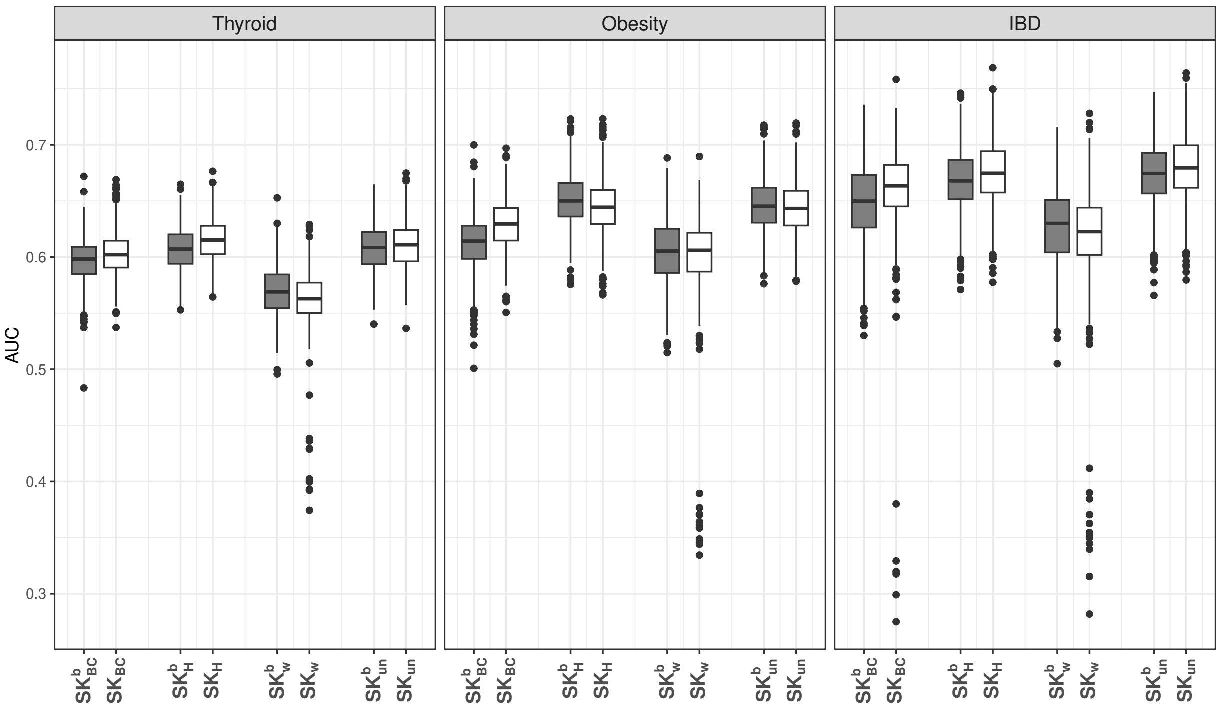

Figure S8: AGP results of box plots of AUCs for the 8 single kernel models from test sets across 1,000 50/50 random splits.

E. Supplementary Tables

Table S1: Simulation results to investigate model performance with covariates with AUC means and 0.025 and 0.975 quantiles (in parentheses) together with kernel weights for covariates in MK-BMC in testing sets over 1,000 simulations. Here signal OTUs are phylogenetically-related (57 signal OTUs with a total abundance of 10.38%) or -unrelated (9 signal OTUs with a total abundance of 11.4%).

	Signal OTUs are Phylogenetically-Related			Signal OTUs are Phylogenetically-Unrelated		
β_{cov}	1.5	2	2.5	1.5	2	2.5
Oracle	0.865 (0.832, 0.892)	0.874 (0.843, 0.901)	0.885 (0.855, 0.912)	0.866 (0.836, 0.894)	0.875 (0.843, 0.903)	0.886 (0.856, 0.913)
RF	0.758 (0.707, 0.800)	0.777 (0.732, 0.815)	0.798 (0.758, 0.836)	**0.770 (0.727, 0.812)**	**0.787 (0.744, 0.827)**	0.807 (0.767, 0.845)
PAAM-RF	0.808 (0.767, 0.845)	0.802 (0.761, 0.839)	0.800 (0.756, 0.838)	0.748 (0.701, 0.793)	0.751 (0.701, 0.794)	0.758 (0.703, 0.805)
SK_{cov}	0.618 (0.576, 0.660)	0.656 (0.616, 0.696)	0.695 (0.654, 0.732)	0.618 (0.576, 0.660)	0.657 (0.618, 0.698)	0.694 (0.652, 0.734)
SK^b_{BC}	0.743 (0.697, 0.785)	0.734 (0.691, 0.776)	0.723 (0.679, 0.765)	0.783 (0.732, 0.828)	0.770 (0.716, 0.815)	0.755 (0.703, 0.802)
SK^b_H	0.529 (0.472, 0.582)	0.526 (0.467, 0.583)	0.523 (0.471, 0.579)	0.531 (0.475, 0.587)	0.528 (0.466, 0.586)	0.525 (0.463, 0.584)
SK^b_w	0.814 (0.774, 0.850)	0.801 (0.764, 0.836)	0.786 (0.745, 0.822)	0.734 (0.675, 0.784)	0.722 (0.657, 0.775)	0.707 (0.640, 0.761)
SK^b_{un}	0.533 (0.478, 0.588)	0.530 (0.473, 0.585)	0.528 (0.473, 0.582)	0.521 (0.469, 0.573)	0.520 (0.467, 0.571)	0.517 (0.467, 0.573)
$SK^b_{BC} + SK_{cov}$	0.748 (0.699, 0.797)	0.772 (0.728, 0.811)	0.802 (0.750, 0.839)	0.755 (0.703, 0.798)	0.785 (0.677, 0.829)	**0.819 (0.778, 0.855)**
$SK^b_H + SK_{cov}$	0.632 (0.581, 0.678)	0.670 (0.622, 0.714)	0.707 (0.662, 0.750)	0.634 (0.584, 0.681)	0.671 (0.622, 0.718)	0.709 (0.663, 0.753)
$SK^b_w + SK_{cov}$	**0.821 (0.757, 0.865)**	0.811 (0.767, 0.860)	**0.832 (0.747, 0.868)**	0.734 (0.680, 0.776)	0.764 (0.678, 0.809)	0.798 (0.756, 0.837)
$SK^b_{un} + SK_{cov}$	0.633 (0.583, 0.682)	0.671 (0.623, 0.716)	0.709 (0.663, 0.754)	0.629 (0.582, 0.676)	0.667 (0.623, 0.716)	0.704 (0.657, 0.749)
MK-BMC	0.816 (0.766, 0.857)	**0.813 (0.759, 0.854)**	0.821 (0.688, 0.864)	0.747 (0.701, 0.788)	0.778 (0.736, 0.819)	0.809 (0.770, 0.846)
5 kernel weights for the 4 kernels for microbiome and 1 kernel for the covariate in MK-BMC						
K^b_{BC}	0.146	0.172	0.108	0.200	0.179	0.168
K^b_H	0.087	0.160	0.105	0.178	0.174	0.165
K^b_w	0.539	0.232	0.110	0.182	0.177	0.166
K^b_{un}	0.086	0.160	0.105	0.176	0.173	0.164
K_{cov}	0.142	0.276	0.572	0.264	0.296	0.336

Table S2: Sensitivity means and 0.025 and 0.975 quantiles (in parentheses) in test sets over 1000 simulations for data generated with $\beta = 2, n = 500$.

Simulation Scenario I: phylogenetically-related clustered OTUs				
Model (Association)	A (Abundance)	A (Abundance)	B (Presence/Absence)	B (Presence/Absence)
OTU proportion (number)	10.38% (57)	4.91% (53)	4.59% (29)	1.43% (25)
Oracle	0.736 (0.684, 0.788)	0.705 (0.648, 0.756)	0.677 (0.616, 0.736)	0.866 (0.820, 0.908)
Mdeep	0.718 (0.644, 0.784)	0.683 (0.612, 0.756)	0.524 (0.440, 0.604)	0.533 (0.448, 0.612)
RF	0.668 (0.588, 0.740)	0.638 (0.568, 0.716)	0.638 (0.560, 0.712)	0.650 (0.572, 0.728)
PAAM-RF	0.731 (0.648, 0.800)	0.724 (0.648, 0.796)	0.648 (0.564, 0.720)	0.753 (0.672, 0.820)
SK^b_{BC}	0.556 (0.476, 0.636)	0.416 (0.336, 0.504)	0.367 (0.060, 0.784)	0.316 (0.036, 0.780)
SK^b_H	0.510 (0.184, 0.872)	0.443 (0.152, 0.776)	0.443 (0.240, 0.628)	0.308 (0.128, 0.452)
SK^b_w	0.752 (0.680, 0.824)	**0.725 (0.648, 0.80)**	0.438 (0.232, 0.688)	0.562 (0.452, 0.664)
SK^b_{un}	0.527 (0.260, 0.800)	0.568 (0.292, 0.808)	**0.779 (0.608, 0.860)**	**0.948 (0.888, 0.984)**
MK-BMC	**0.750 (0.668, 0.824)**	0.667 (0.556, 0.780)	0.660 (0.488, 0.828)	0.786 (0.540, 0.980)
Simulation Scenario II: not phylogenetically-related subset of OTUs				
Model (Association)	A (Abundance)	A (Abundance)	B (Presence/Absence)	B (Presence/Absence)
OTU proportion (number)	11.41% (9)	4.77% (9)	10.91% (9)	2.26% (9)
Oracle	0.740 (0.688, 0.788)	0.704 (0.652, 0.756)	0.908 (0.640, 0.948)	0.824 (0.772, 0.876)
Mdeep	0.716 (0.640, 0.784)	0.668 (0.588, 0.740)	0.554 (0.476, 0.632)	0.552 (0.464, 0.632)
RF	0.705 (0.632, 0.776)	0.701 (0.624, 0.772)	0.680 (0.608, 0.744)	0.711 (0.640, 0.784)
PAAM-RF	0.702 (0.624, 0.772)	0.682 (0.600, 0.752)	0.670 (0.592, 0.740)	0.651 (0.576, 0.724)
SK^b_{BC}	0.295 (0.176, 0.420)	0.169 (0.072, 0.304)	0.505 (0.232, 0.852)	0.301 (0.068, 0.688)
SK^b_H	**0.768 (0.416, 0.964)**	0.640 (0.392, 0.844)	0.810 (0.668, 0.912)	0.508 (0.396, 0.620)
SK^b_w	0.554 (0.404, 0.700)	0.339 (0.184, 0.516)	0.589 (0.336, 0.828)	0.453 (0.180, 0.736)
SK^b_{un}	0.624 (0.316, 0.900)	**0.734 (0.528, 0.884)**	**0.922 (0.832, 0.984)**	**0.836 (0.724, 0.916)**
MK-BMC	0.474 (0.316, 0.636)	0.275 (0.124, 0.460)	0.831 (0.696, 0.924)	0.549 (0.412, 0.696)
Simulation Scenario III: mixture of Scenario I and Scenario II				
Association	$P_{phy} + P_{unphy}$	$P_{phy} + A_{unphy}$	$A_{phy} + P_{unphy}$	$A_{unphy} + P_{unphy}$
OTU proportion (number)	4.59%+10.91% (29+9)	1.43%+11.41% (25+9)	10.38%+2.26% (57+9)	11.41%+2.26% (9+9)
Oracle	0.753 (0.696, 0.812)	0.809 (0.764, 0.852)	0.812 (0.764, 0.856)	0.814 (0.764, 0.856)
Mdeep	0.544 (0.460, 0.628)	0.664 (0.588, 0.732)	0.661 (0.584, 0.732)	0.672 (0.604, 0.740)
RF	0.662 (0.584, 0.732)	0.695 (0.624, 0.764)	0.713 (0.640, 0.780)	0.726 (0.652, 0.796)
PAAM-RF	0.652 (0.580, 0.724)	0.725 (0.652, 0.796)	0.666 (0.584, 0.748)	0.674 (0.596, 0.744)
SK^b_{BC}	0.464 (0.132, 0.856)	0.293 (0.160, 0.460)	0.499 (0.412, 0.592)	0.297 (0.160, 0.476)
SK^b_H	0.701 (0.532, 0.840)	0.367 (0.168, 0.572)	0.470 (0.332, 0.608)	0.529 (0.384, 0.676)
SK^b_w	0.516 (0.264, 0.788)	0.555 (0.400, 0.712)	0.674 (0.596, 0.748)	0.526 (0.348, 0.704)
SK^b_{un}	**0.874 (0.708, 0.972)**	**0.903 (0.792, 0.964)**	**0.762 (0.624, 0.872)**	**0.777 (0.632, 0.884)**
MK-BMC	0.781 (0.612, 0.904)	0.597 (0.388, 0.792)	0.618 (0.532, 0.712)	0.534 (0.360, 0.684)

Table S3: Specificity means and 0.025 and 0.975 quantiles (in parentheses) in test sets over 1000 simulations for data generated with $\beta = 2, n = 500$.

Simulation Scenario I: phylogenetically-related clustered OTUs				
Model (Association)	A (Abundance)	A (Abundance)	B (Presence/Absence)	B (Presence/Absence)
OTU proportion (number)	10.38% (57)	4.91% (53)	4.59% (29)	1.43% (25)
Oracle	0.814 (0.768, 0.860)	0.832 (0.788, 0.876)	0.878 (0.832, 0.920)	0.682 (0.616, 0.74)
Mdeep	0.731 (0.656, 0.804)	0.738 (0.660, 0.804)	0.535 (0.452, 0.620)	0.554 (0.464, 0.652)
RF	0.704 (0.632, 0.780)	0.680 (0.600, 0.756)	0.656 (0.584, 0.724)	0.655 (0.576, 0.728)
PAAM-RF	0.777 (0.704, 0.848)	0.780 (0.700, 0.848)	0.637 (0.552, 0.712)	0.626 (0.548, 0.700)
SK^b_{BC}	**0.806 (0.736, 0.872)**	**0.868 (0.808, 0.928)**	0.722 (0.292, 0.976)	0.760 (0.284, 0.992)
SK^b_H	0.531 (0.148, 0.848)	0.619 (0.284, 0.888)	**0.834 (0.720, 0.956)**	**0.913 (0.800, 0.988)**
SK^b_w	0.752 (0.680, 0.824)	0.782 (0.708, 0.852)	0.727 (0.448, 0.912)	0.759 (0.672, 0.836)
SK^b_{un}	0.523 (0.256, 0.772)	0.504 (0.264, 0.756)	0.579 (0.484, 0.716)	0.349 (0.252, 0.476)
MK-BMC	0.753 (0.680, 0.828)	0.817 (0.728, 0.888)	0.689 (0.524, 0.820)	0.569 (0.264, 0.832)
Simulation Scenario II: not phylogenetically-related subset of OTUs				
Model (Association)	A (Abundance)	A (Abundance)	B (Presence/Absence)	B (Presence/Absence)
OTU proportion (number)	11.41% (9)	4.77% (9)	10.91% (9)	2.26% (9)
Oracle	0.812 (0.768, 0.856)	0.833 (0.788, 0.876)	0.613 (0.540, 0.900)	0.750 (0.684, 0.808)
Mdeep	0.697 (0.620, 0.768)	0.693 (0.616, 0.768)	0.543 (0.456, 0.624)	0.559 (0.476, 0.644)
RF	0.679 (0.604, 0.752)	0.679 (0.604, 0.752)	**0.712 (0.64, 0.776)**	0.673 (0.592, 0.748)
PAAM-RF	0.682 (0.608, 0.756)	0.677 (0.600, 0.752)	0.692 (0.620, 0.764)	0.639 (0.560, 0.716)
SK^b_{BC}	**0.960 (0.900, 0.996)**	**0.985 (0.952, 1.000)**	0.658 (0.260, 0.892)	0.836 (0.472, 0.988)
SK^b_H	0.269 (0.044, 0.628)	0.495 (0.240, 0.720)	0.590 (0.468, 0.692)	**0.879 (0.764, 0.952)**
SK^b_w	0.792 (0.664, 0.900)	0.916 (0.792, 0.980)	0.524 (0.264, 0.776)	0.657 (0.348, 0.896)
SK^b_{un}	0.404 (0.132, 0.712)	0.373 (0.196, 0.580)	0.329 (0.192, 0.476)	0.498 (0.392, 0.624)
MK-BMC	0.873 (0.760, 0.956)	0.958 (0.872, 1.000)	0.551 (0.400, 0.672)	0.851 (0.716, 0.944)
Simulation Scenario III: mixture of Scenario I and Scenario II				
Association	$P_{phy} + P_{unphy}$	$P_{phy} + A_{unphy}$	$A_{phy} + P_{unphy}$	$A_{unphy} + P_{unphy}$
OTU proportion (number)	4.59%+10.91% (29+9)	1.43%+11.41% (25+9)	10.38%+2.26% (57+9)	11.41%+2.26% (9+9)
Oracle	0.899 (0.856, 0.936)	0.843 (0.800, 0.884)	0.840 (0.796, 0.884)	0.837 (0.796, 0.880)
Mdeep	0.539 (0.452, 0.620)	0.653 (0.568, 0.728)	0.669 (0.592, 0.740)	0.652 (0.572, 0.724)
RF	**0.692 (0.616, 0.760)**	0.672 (0.600, 0.744)	0.689 (0.620, 0.760)	0.680 (0.604, 0.752)
PAAM-RF	0.669 (0.592, 0.740)	0.669 (0.596, 0.744)	0.735 (0.640, 0.808)	0.661 (0.584, 0.736)
SK^b_{BC}	0.661 (0.244, 0.936)	**0.918 (0.820, 0.980)**	0.773 (0.696, 0.848)	**0.916 (0.800, 0.980)**
SK^b_H	0.685 (0.540, 0.840)	0.828 (0.648, 0.956)	**0.819 (0.688, 0.924)**	0.774 (0.636, 0.888)
SK^b_w	0.620 (0.324, 0.852)	0.767 (0.636, 0.868)	0.695 (0.612, 0.772)	0.724 (0.548, 0.860)
SK^b_{un}	0.425 (0.240, 0.616)	0.327 (0.220, 0.484)	0.485 (0.348, 0.628)	0.461 (0.320, 0.612)
MK-BMC	0.608 (0.440, 0.768)	0.795 (0.596, 0.932)	0.773 (0.692, 0.848)	0.850 (0.708, 0.948)

Table S4: AUC means and 0.025 and 0.975 quantiles (in parentheses) in test sets over 1000 simulations for data generated with $\beta = 2, n = 200$.

Simulation Scenario I: phylogenetically-related clustered OTUs				
Model (Association)	A (Abundance)	A (Abundance)	B (Presence/Absence)	B (Presence/Absence)
OTU proportion (number)	10.38% (57)	4.91% (53)	4.59% (29)	1.43% (25)
Oracle	0.858 (0.808, 0.904)	0.850 (0.795, 0.899)	0.849 (0.795, 0.895)	0.851 (0.804, 0.898)
Mdeep	0.778 (0.710, 0.840)	0.739 (0.660, 0.810)	0.520 (0.444, 0.597)	0.522 (0.442, 0.598)
RF	0.699 (0.614, 0.774)	0.659 (0.578, 0.740)	0.619 (0.529, 0.705)	0.620 (0.538, 0.706)
PAAM-RF	0.814 (0.753, 0.869)	0.802 (0.742, 0.859)	0.609 (0.518, 0.706)	0.721 (0.647, 0.792)
SK^b_{BC}	0.755 (0.685, 0.816)	0.705 (0.630, 0.774)	0.540 (0.451, 0.623)	0.537 (0.455, 0.621)
SK^b_H	0.519 (0.439, 0.602)	0.532 (0.444, 0.614)	0.681 (0.590, 0.765)	0.680 (0.594, 0.759)
SK^b_w	**0.833 (0.777, 0.883)**	**0.830 (0.774, 0.882)**	0.574 (0.476, 0.669)	0.702 (0.591, 0.789)
SK^b_{un}	0.522 (0.449, 0.605)	0.534 (0.447, 0.617)	**0.736 (0.657, 0.808)**	0.750 (0.673, 0.818)
MK-BMC	0.814 (0.752, 0.869)	0.813 (0.748, 0.865)	0.715 (0.630, 0.796)	**0.751 (0.672, 0.822)**
Simulation Scenario II: not phylogenetically-related subset of OTUs				
Model (Association)	A (Abundance)	A (Abundance)	B (Presence/Absence)	B (Presence/Absence)
OTU proportion (number)	11.41% (9)	4.77% (9)	10.91% (9)	2.26% (9)
Oracle	0.858 (0.811, 0.903)	0.850 (0.797, 0.899)	0.852 (0.806, 0.895)	0.856 (0.806, 0.898)
Mdeep	0.726 (0.645, 0.796)	0.660 (0.577, 0.742)	0.542 (0.453, 0.626)	0.541 (0.460, 0.627)
RF	0.696 (0.616, 0.772)	0.681 (0.601, 0.761)	0.688 (0.601, 0.765)	0.685 (0.601, 0.762)
PAAM-RF	0.701 (0.623, 0.777)	0.662 (0.578, 0.746)	0.662 (0.574, 0.746)	0.611 (0.521, 0.697)
SK^b_{BC}	**0.760 (0.677, 0.837)**	**0.732 (0.625, 0.822)**	0.583 (0.485, 0.673)	0.577 (0.488, 0.666)
SK^b_H	0.519 (0.439, 0.598)	0.564 (0.474, 0.648)	**0.761 (0.685, 0.828)**	**0.765 (0.683, 0.834)**
SK^b_w	0.695 (0.601, 0.784)	0.679 (0.575, 0.773)	0.548 (0.457, 0.633)	0.545 (0.455, 0.637)
SK^b_{un}	0.512 (0.435, 0.597)	0.555 (0.467, 0.639)	0.719 (0.631, 0.792)	0.717 (0.632, 0.793)
MK-BMC	0.708 (0.616, 0.792)	0.675 (0.575, 0.762)	0.751 (0.671, 0.824)	0.754 (0.668, 0.826)
Simulation Scenario III: mixture of Scenario I and Scenario II				
Association	$P_{phy} + P_{unphy}$	$P_{phy} + A_{unphy}$	$A_{phy} + P_{unphy}$	$A_{unphy} + P_{unphy}$
OTU proportion (number)	4.59%+10.91% (29+9)	1.43%+11.41% (25+9)	10.38%+2.26% (57+9)	11.41%+2.26% (9+9)
Oracle	0.906 (0.865, 0.942)	0.910 (0.867, 0.944)	0.911 (0.873, 0.944)	0.910 (0.871, 0.945)
Mdeep	0.535 (0.451, 0.622)	0.669 (0.582, 0.744)	0.703 (0.620, 0.777)	0.676 (0.598, 0.756)
RF	0.663 (0.579, 0.745)	0.676 (0.595, 0.755)	0.705 (0.626, 0.782)	0.703 (0.618, 0.779)
PAAM-RF	0.635 (0.546, 0.725)	0.722 (0.640, 0.796)	0.744 (0.669, 0.811)	0.666 (0.574, 0.747)
SK^b_{BC}	0.563 (0.474, 0.650)	0.681 (0.596, 0.765)	0.699 (0.618, 0.770)	0.688 (0.598, 0.768)
SK^b_H	0.731 (0.650, 0.808)	0.609 (0.514, 0.694)	0.676 (0.589, 0.760)	0.678 (0.588, 0.759)
SK^b_w	0.563 (0.469, 0.655)	0.691 (0.588, 0.778)	0.750 (0.677, 0.817)	0.639 (0.542, 0.725)
SK^b_{un}	0.728 (0.645, 0.803)	0.669 (0.582, 0.753)	0.642 (0.557, 0.726)	0.641 (0.549, 0.723)
MK-BMC	**0.735 (0.653, 0.812)**	**0.729 (0.638, 0.807)**	**0.779 (0.709, 0.845)**	**0.726 (0.632, 0.810)**

Table S5: Sensitivity means and 0.025 and 0.975 quantiles (in parentheses) in test sets over 1000 simulations for data generated with $\beta = 2, n = 200$.

Simulation Scenario I: phylogenetically-related clustered OTUs				
Model (Association)	A (Abundance)	A (Abundance)	B (Presence/Absence)	B (Presence/Absence)
OTU proportion (number)	10.38% (57)	4.91% (53)	4.59% (29)	1.43% (25)
Oracle	0.739 (0.660, 0.820)	0.708 (0.630, 0.790)	0.676 (0.570, 0.770)	0.853 (0.610, 0.930)
Mdeep	0.700 (0.580, 0.810)	0.638 (0.520, 0.760)	0.519 (0.280, 0.770)	0.514 (0.280, 0.770)
RF	0.643 (0.530, 0.750)	0.606 (0.480, 0.720)	0.570 (0.450, 0.690)	0.570 (0.460, 0.680)
PAAM-RF	0.722 (0.610, 0.820)	0.701 (0.580, 0.810)	0.575 (0.450, 0.690)	0.694 (0.570, 0.810)
SK^b_{BC}	0.569 (0.430, 0.690)	0.438 (0.290, 0.600)	0.469 (0.120, 0.840)	0.435 (0.070, 0.850)
SK^b_H	0.509 (0.160, 0.840)	0.462 (0.150, 0.840)	0.427 (0.170, 0.720)	0.329 (0.100, 0.580)
SK^b_w	**0.755 (0.650, 0.850)**	**0.718 (0.590, 0.830)**	0.480 (0.220, 0.760)	0.553 (0.350, 0.740)
SK^b_{un}	0.502 (0.220, 0.790)	0.522 (0.250, 0.790)	**0.748 (0.530, 0.920)**	**0.903 (0.710, 0.990)**
MK-BMC	0.694 (0.560, 0.810)	0.646 (0.510, 0.780)	0.604 (0.350, 0.850)	0.679 (0.380, 0.960)
Simulation Scenario II: not phylogenetically-related subset of OTUs				
Model (Association)	A (Abundance)	A (Abundance)	B (Presence/Absence)	B (Presence/Absence)
OTU proportion (number)	11.41% (9)	4.77% ()	10.91% (9)	2.26% (9)
Oracle	0.738 (0.650, 0.810)	0.707 (0.620, 0.790)	0.876 (0.580, 0.970)	0.827 (0.750, 0.900)
Mdeep	0.657 (0.530, 0.780)	0.592 (0.440, 0.740)	0.536 (0.320, 0.740)	0.520 (0.290, 0.730)
RF	0.651 (0.530, 0.760)	0.632 (0.510, 0.740)	0.632 (0.510, 0.750)	0.639 (0.520, 0.750)
PAAM-RF	0.646 (0.530, 0.760)	0.616 (0.500, 0.730)	0.614 (0.500, 0.720)	0.581 (0.460, 0.700)
SK^b_{BC}	0.392 (0.190, 0.620)	0.275 (0.090, 0.530)	0.533 (0.180, 0.890)	0.439 (0.090, 0.850)
SK^b_H	**0.663 (0.290, 0.950)**	0.592 (0.260, 0.870)	0.778 (0.600, 0.940)	0.501 (0.310, 0.700)
SK^b_w	0.563 (0.350, 0.780)	0.407 (0.190, 0.650)	0.565 (0.250, 0.870)	0.499 (0.200, 0.800)
SK^b_{un}	0.562 (0.270, 0.850)	**0.651 (0.360, 0.880)**	**0.887 (0.710, 0.990)**	**0.772 (0.590, 0.910)**
MK-BMC	0.536 (0.290, 0.780)	0.458 (0.210, 0.720)	0.808 (0.620, 0.950)	0.559 (0.350, 0.760)
Simulation Scenario III: mixture of Scenario I and Scenario II				
Association	$P_{phy} + P_{unphy}$	$P_{phy} + A_{unphy}$	$A_{phy} + P_{unphy}$	$A_{unphy} + P_{unphy}$
OTU proportion (number)	4.59%+10.91% (29+9)	1.43%+11.41% (25+9)	10.38%+2.26% (57+9)	11.41%+2.26% (9+9)
Oracle	0.755 (0.660, 0.850)	0.810 (0.740, 0.880)	0.812 (0.740, 0.880)	0.814 (0.730, 0.890)
Mdeep	0.525 (0.310, 0.740)	0.616 (0.480, 0.750)	0.641 (0.510, 0.760)	0.620 (0.470, 0.760)
RF	0.607 (0.490, 0.730)	0.625 (0.510, 0.740)	0.656 (0.530, 0.760)	0.660 (0.540, 0.770)
PAAM-RF	0.594 (0.470, 0.710)	0.669 (0.550, 0.780)	0.658 (0.530, 0.770)	0.620 (0.500, 0.730)
SK^b_{BC}	0.508 (0.150, 0.880)	0.400 (0.170, 0.670)	0.520 (0.370, 0.670)	0.412 (0.180, 0.690)
SK^b_H	0.657 (0.370, 0.870)	0.433 (0.150, 0.790)	0.468 (0.230, 0.740)	0.547 (0.290, 0.790)
SK^b_w	0.531 (0.260, 0.830)	0.560 (0.340, 0.790)	0.675 (0.540, 0.790)	0.549 (0.320, 0.770)
SK^b_{un}	**0.851 (0.640, 0.980)**	**0.813 (0.530, 0.960)**	**0.694 (0.480, 0.870)**	**0.717 (0.510, 0.890)**
MK-BMC	0.734 (0.510, 0.930)	0.589 (0.310, 0.850)	0.635 (0.480, 0.780)	0.573 (0.330, 0.800)

Table S6: Specificity means and 0.025 and 0.975 quantiles (in parentheses) in test sets over 1000 simulations for data generated with $\beta = 2, n = 200$.

Simulation Scenario I: phylogenetically-related clustered OTUs				
Model (Association)	A (Abundance)	A (Abundance)	B (Presence/Absence)	B (Presence/Absence)
OTU proportion (number)	10.38% (57)	4.91% (53)	4.59% (29)	1.43% (25)
Oracle	0.813 (0.740, 0.880)	0.831 (0.760, 0.890)	0.878 (0.800, 0.940)	0.698 (0.600, 0.920)
Mdeep	0.709 (0.590, 0.820)	0.718 (0.590, 0.830)	0.511 (0.270, 0.740)	0.514 (0.250, 0.750)
RF	0.652 (0.530, 0.770)	0.625 (0.510, 0.740)	0.598 (0.480, 0.720)	0.600 (0.470, 0.720)
PAAM-RF	0.764 (0.650, 0.860)	0.769 (0.660, 0.870)	0.578 (0.450, 0.710)	0.639 (0.530, 0.750)
SK^b_{BC}	**0.795 (0.680, 0.890)**	0.847 (0.710, 0.940)	0.582 (0.180, 0.920)	0.609 (0.170, 0.950)
SK^b_H	0.514 (0.160, 0.860)	0.576 (0.190, 0.890)	**0.797 (0.520, 0.960)**	**0.865 (0.680, 0.990)**
SK^b_w	0.747 (0.630, 0.850)	0.784 (0.670, 0.890)	0.617 (0.300, 0.880)	0.727 (0.470, 0.870)
SK^b_{un}	0.527 (0.240, 0.800)	0.523 (0.250, 0.790)	0.582 (0.310, 0.770)	0.402 (0.240, 0.640)
MK-BMC	0.773 (0.650, 0.870)	**0.819 (0.700, 0.920)**	0.695 (0.440, 0.870)	0.661 (0.320, 0.880)
Simulation Scenario II: not phylogenetically-related subset of OTUs				
Model (Association)	A (Abundance)	A (Abundance)	B (Presence/Absence)	B (Presence/Absence)
OTU proportion (number)	11.41% (9)	4.77% (9)	10.91% (9)	2.26% (9)
Oracle	0.811 (0.730, 0.880)	0.835 (0.770, 0.900)	0.651 (0.520, 0.940)	0.749 (0.660, 0.840)
Mdeep	0.670 (0.540, 0.800)	0.635 (0.480, 0.780)	0.523 (0.310, 0.740)	0.536 (0.310, 0.750)
RF	0.631 (0.510, 0.740)	0.627 (0.510, 0.750)	**0.640 (0.520, 0.750)**	0.628 (0.510, 0.750)
PAAM-RF	0.645 (0.530, 0.750)	0.616 (0.500, 0.720)	0.620 (0.500, 0.750)	0.575 (0.450, 0.680)
SK^b_{BC}	**0.891 (0.730, 0.990)**	**0.930 (0.740, 1.000)**	0.570 (0.170, 0.890)	0.651 (0.220, 0.960)
SK^b_H	0.360 (0.070, 0.740)	0.491 (0.170, 0.800)	0.568 (0.340, 0.740)	**0.835 (0.670, 0.960)**
SK^b_w	0.702 (0.480, 0.880)	0.806 (0.560, 0.960)	0.497 (0.180, 0.790)	0.559 (0.230, 0.860)
SK^b_{un}	0.454 (0.150, 0.750)	0.422 (0.170, 0.690)	0.336 (0.130, 0.560)	0.513 (0.330, 0.690)
MK-BMC	0.743 (0.460, 0.930)	0.766 (0.480, 0.950)	0.512 (0.270, 0.710)	0.782 (0.600, 0.940)
Simulation Scenario III: mixture of Scenario I and Scenario II				
Association	$P_{phy} + P_{unphy}$	$P_{phy} + A_{unphy}$	$A_{phy} + P_{unphy}$	$A_{unphy} + P_{unphy}$
OTU proportion (number)	4.59%+10.91% (29+9)	1.43%+11.41% (25+9)	10.38%+2.26% (57+9)	11.41%+2.26% (9+9)
Oracle	0.893 (0.820, 0.950)	0.842 (0.770, 0.910)	0.840 (0.770, 0.910)	0.839 (0.770, 0.900)
Mdeep	0.522 (0.300, 0.730)	0.623 (0.470, 0.760)	0.652 (0.520, 0.770)	0.630 (0.480, 0.780)
RF	0.627 (0.520, 0.730)	0.626 (0.510, 0.740)	0.640 (0.520, 0.750)	0.632 (0.510, 0.750)
PAAM-RF	0.598 (0.480, 0.710)	0.654 (0.540, 0.770)	0.706 (0.580, 0.820)	0.617 (0.490, 0.730)
SK^b_{BC}	0.568 (0.120, 0.910)	**0.812 (0.570, 0.970)**	0.756 (0.600, 0.880)	**0.809 (0.510, 0.970)**
SK^b_H	**0.655 (0.400, 0.890)**	0.705 (0.340, 0.930)	**0.757 (0.500, 0.930)**	0.693 (0.400, 0.900)
SK^b_w	0.549 (0.240, 0.830)	0.697 (0.440, 0.870)	0.690 (0.550, 0.810)	0.639 (0.370, 0.850)
SK^b_{un}	0.406 (0.180, 0.640)	0.410 (0.210, 0.670)	0.497 (0.290, 0.700)	0.465 (0.250, 0.680)
MK-BMC	0.584 (0.300, 0.810)	0.719 (0.450, 0.920)	0.756 (0.630, 0.870)	0.733 (0.500, 0.920)

Table S7: AUC means and 0.025 and 0.975 quantiles (in parentheses) in test sets over 1000 simulations for data generated with $\beta = 2, n = 100$.

Simulation Scenario I: phylogenetically-related clustered OTUs				
Model (Association)	A (Abundance)	A (Abundance)	B (Presence/Absence)	B (Presence/Absence)
OTU proportion (number)	10.38% (57)	4.91% (53)	4.59% (29)	1.43% (25)
Oracle	0.857 (0.782, 0.920)	0.850 (0.767, 0.920)	0.850 (0.780, 0.911)	0.852 (0.777, 0.913)
Mdeep	0.744 (0.623, 0.842)	0.662 (0.528, 0.772)	0.511 (0.402, 0.618)	0.511 (0.392, 0.635)
RF	0.653 (0.531, 0.770)	0.618 (0.501, 0.723)	0.575 (0.450, 0.691)	0.574 (0.462, 0.693)
PAAM-RF	0.795 (0.701, 0.876)	0.783 (0.684, 0.866)	0.564 (0.447, 0.679)	0.679 (0.555, 0.793)
SK^b_{BC}	0.746 (0.643, 0.836)	0.684 (0.554, 0.791)	0.522 (0.400, 0.643)	0.521 (0.400, 0.634)
SK^b_{H}	0.513 (0.395, 0.628)	0.520 (0.402, 0.631)	0.624 (0.486, 0.744)	0.621 (0.491, 0.745)
SK^b_{w}	**0.827 (0.744, 0.904)**	**0.820 (0.734, 0.896)**	0.546 (0.417, 0.672)	0.643 (0.483, 0.776)
SK^b_{un}	0.513 (0.402, 0.620)	0.523 (0.409, 0.636)	**0.694 (0.570, 0.800)**	**0.703 (0.584, 0.811)**
MK-BMC	0.802 (0.711, 0.883)	0.784 (0.676, 0.872)	0.655 (0.515, 0.770)	0.684 (0.548, 0.807)
Simulation Scenario II: not phylogenetically-related subset of OTUs				
Model (Association)	A (Abundance)	A (Abundance)	B (Presence/Absence)	B (Presence/Absence)
OTU proportion (number)	11.41% (9)	4.77% (9)	10.91% (9)	2.26% (9)
Oracle	0.860 (0.786, 0.926)	0.850 (0.769, 0.918)	0.851 (0.778, 0.912)	0.855 (0.790, 0.916)
Mdeep	0.696 (0.579, 0.804)	0.619 (0.508, 0.733)	0.530 (0.414, 0.638)	0.529 (0.411, 0.640)
RF	0.646 (0.538, 0.756)	0.629 (0.509, 0.743)	0.632 (0.515, 0.742)	0.631 (0.515, 0.739)
PAAM-RF	0.650 (0.532, 0.768)	0.607 (0.486, 0.722)	0.603 (0.482, 0.719)	0.567 (0.451, 0.693)
SK^b_{BC}	**0.712 (0.592, 0.825)**	**0.664 (0.527, 0.79)**	0.555 (0.444, 0.674)	0.550 (0.431, 0.670)
SK^b_{H}	0.510 (0.399, 0.623)	0.545 (0.430, 0.664)	**0.707 (0.588, 0.817)**	**0.707 (0.584, 0.810)**
SK^b_{w}	0.646 (0.522, 0.773)	0.618 (0.495, 0.737)	0.533 (0.416, 0.641)	0.531 (0.414, 0.647)
SK^b_{un}	0.507 (0.399, 0.612)	0.538 (0.420, 0.654)	0.657 (0.536, 0.771)	0.665 (0.536, 0.782)
MK-BMC	0.620 (0.468, 0.757)	0.600 (0.472, 0.720)	0.693 (0.578, 0.799)	0.695 (0.565, 0.802)
Simulation Scenario III: mixture of Scenario I and Scenario II				
Association	$P_{phy} + P_{unphy}$	$P_{phy} + A_{unphy}$	$A_{phy} + P_{unphy}$	$A_{unphy} + P_{unphy}$
OTU proportion (number)	4.59%+10.91% (29+9)	1.43%+11.41% (25+9)	10.38%+2.26% (57+9)	11.41%+2.26% (9+9)
Oracle	0.906 (0.845, 0.956)	0.911 (0.854, 0.959)	0.910 (0.855, 0.958)	0.910 (0.853, 0.959)
Mdeep	0.525 (0.410, 0.636)	0.639 (0.522, 0.754)	0.676 (0.550, 0.783)	0.643 (0.526, 0.760)
RF	0.613 (0.491, 0.724)	0.628 (0.510, 0.736)	0.658 (0.538, 0.770)	0.653 (0.539, 0.761)
PAAM-RF	0.586 (0.464, 0.709)	**0.671 (0.547, 0.787)**	0.717 (0.602, 0.816)	0.624 (0.505, 0.739)
SK^b_{BC}	0.544 (0.423, 0.665)	0.647 (0.519, 0.759)	0.686 (0.575, 0.794)	0.650 (0.523, 0.771)
SK^b_{H}	0.670 (0.555, 0.781)	0.573 (0.451, 0.695)	0.629 (0.503, 0.748)	0.630 (0.502, 0.748)
SK^b_{w}	0.543 (0.429, 0.658)	0.643 (0.502, 0.768)	0.743 (0.643, 0.834)	0.604 (0.474, 0.727)
SK^b_{un}	**0.677 (0.558, 0.786)**	0.625 (0.493, 0.747)	0.602 (0.483, 0.716)	0.601 (0.478, 0.716)
MK-BMC	0.675 (0.558, 0.789)	0.653 (0.518, 0.782)	**0.751 (0.638, 0.846)**	**0.661 (0.540, 0.770)**

Table S8: Sensitivity means and 0.025 and 0.975 quantiles (in parentheses) in test sets over 1000 simulations for data generated with $\beta = 2, n = 100$.

Simulation Scenario I: phylogenetically-related clustered OTUs				
Model (Association)	A (Abundance)	A (Abundance)	B (Presence/Absence)	B (Presence/Absence)
OTU proportion (number)	10.38% (57)	4.91% (53)	4.59% (29)	1.43% (25)
Oracle	0.737 (0.620, 0.840)	0.706 (0.580, 0.820)	0.677 (0.540, 0.820)	0.831 (0.540, 0.940)
Mdeep	0.679 (0.180, 0.980)	0.562 (0.020, 1.000)	0.505 (0.000, 1.000)	0.490 (0.000, 1.000)
RF	0.607 (0.440, 0.760)	0.577 (0.420, 0.740)	0.540 (0.380, 0.700)	0.532 (0.360, 0.700)
PAAM-RF	0.704 (0.540, 0.860)	0.686 (0.520, 0.840)	0.542 (0.380, 0.700)	0.637 (0.460, 0.800)
SK^b_{BC}	0.579 (0.400, 0.780)	0.475 (0.240, 0.760)	0.491 (0.120, 0.900)	0.470 (0.100, 0.880)
SK^b_H	0.494 (0.120, 0.880)	0.484 (0.140, 0.860)	0.459 (0.160, 0.780)	0.364 (0.080, 0.700)
SK^b_w	**0.747 (0.580, 0.90)**	**0.705 (0.520, 0.860)**	0.493 (0.180, 0.820)	0.537 (0.280, 0.800)
SK^b_{un}	0.491 (0.200, 0.780)	0.515 (0.200, 0.800)	**0.708 (0.460, 0.920)**	**0.807 (0.500, 0.980)**
MK-BMC	0.688 (0.500, 0.860)	0.643 (0.420, 0.860)	0.563 (0.260, 0.840)	0.563 (0.240, 0.900)
Simulation Scenario II: not phylogenetically-related subset of OTUs				
Model (Association)	A (Abundance)	A (Abundance)	B (Presence/Absence)	B (Presence/Absence)
OTU proportion (number)	11.41% (9)	4.77% ()	10.91% (9)	2.26% (9)
Oracle	0.743 (0.620, 0.860)	0.707 (0.580, 0.820)	0.849 (0.560, 0.980)	0.825 (0.720, 0.920)
Mdeep	0.595 (0.020, 1.000)	0.543 (0.000, 1.000)	0.543 (0.000, 1.000)	0.501 (0.000, 1.000)
RF	**0.614 (0.460, 0.780)**	0.594 (0.440, 0.760)	0.597 (0.440, 0.760)	0.590 (0.440, 0.740)
PAAM-RF	0.609 (0.440, 0.780)	0.577 (0.420, 0.740)	0.575 (0.420, 0.740)	0.551 (0.400, 0.700)
SK^b_{BC}	0.471 (0.200, 0.780)	0.403 (0.100, 0.780)	0.537 (0.140, 0.900)	0.464 (0.100, 0.880)
SK^b_H	0.607 (0.220, 0.940)	0.570 (0.220, 0.880)	0.734 (0.460, 0.940)	0.493 (0.220, 0.760)
SK^b_w	0.577 (0.300, 0.860)	0.468 (0.180, 0.780)	0.548 (0.240, 0.840)	0.497 (0.180, 0.800)
SK^b_{un}	0.525 (0.220, 0.820)	**0.611 (0.320, 0.880)**	**0.817 (0.560, 0.980)**	**0.715 (0.460, 0.920)**
MK-BMC	0.574 (0.260, 0.860)	0.535 (0.240, 0.840)	0.754 (0.480, 0.940)	0.542 (0.260, 0.820)
Simulation Scenario III: mixture of Scenario I and Senario II				
Association	$P_{phy} + P_{unphy}$	$P_{phy} + A_{unphy}$	$A_{phy} + P_{unphy}$	$A_{unphy} + P_{unphy}$
OTU proportion (number)	4.59%+10.91% (29+9)	1.43%+11.41% (25+9)	10.38%+2.26% (57+9)	11.41%+2.26% (9+9)
Oracle	0.766 (0.6390, 0.90)	0.812 (0.700, 0.920)	0.811 (0.700, 0.920)	0.812 (0.700, 0.900)
Mdeep	0.508 (0.000, 1.000)	0.562 (0.000, 1.000)	0.628 (0.080, 1.000)	0.557 (0.020, 1.000)
RF	0.571 (0.400, 0.740)	0.591 (0.440, 0.740)	0.615 (0.460, 0.780)	0.616 (0.460, 0.760)
PAAM-RF	0.561 (0.380, 0.740)	0.627 (0.480, 0.780)	0.645 (0.480, 0.800)	0.591 (0.440, 0.740)
SK^b_{BC}	0.514 (0.140, 0.920)	0.471 (0.160, 0.820)	0.545 (0.320, 0.800)	0.475 (0.180, 0.820)
SK^b_H	0.612 (0.280, 0.880)	0.465 (0.140, 0.840)	0.471 (0.160, 0.800)	0.553 (0.260, 0.860)
SK^b_w	0.524 (0.220, 0.840)	0.565 (0.280, 0.820)	**0.671 (0.480, 0.840)**	0.557 (0.280, 0.840)
SK^b_{un}	**0.795 (0.540, 0.980)**	**0.707 (0.380, 0.940)**	0.639 (0.360, 0.880)	**0.661 (0.360, 0.90)**
MK-BMC	0.675 (0.360, 0.920)	0.561 (0.240, 0.860)	0.626 (0.340, 0.860)	0.584 (0.300, 0.840)

Table S9: Specificity means and 0.025 and 0.975 quantiles (in parentheses) in test sets over 1000 simulations for data generated with $\beta = 2, n = 100$.

Simulation Scenario I: phylogenetically-related clustered OTUs				
Model (Association)	A (Abundance)	A (Abundance)	B (Presence/Absence)	B (Presence/Absence)
OTU proportion (number)	10.38% (57)	4.91% (53)	4.59% (29)	1.43% (25)
Oracle	0.812 (0.719, 0.900)	0.834 (0.720, 0.920)	0.878 (0.780, 0.960)	0.719 (0.560, 0.960)
Mdeep	0.620 (0.080, 0.980)	0.598 (0.000, 1.000)	0.504 (0.000, 1.000)	0.515 (0.000, 1.000)
RF	0.614 (0.440, 0.780)	0.592 (0.420, 0.760)	0.566 (0.380, 0.720)	0.571 (0.400, 0.740)
PAAM-RF	0.749 (0.580, 0.900)	0.745 (0.580, 0.880)	0.549 (0.380, 0.720)	0.630 (0.460, 0.780)
SK^b_{BC}	**0.778 (0.560, 0.920)**	**0.781 (0.440, 0.960)**	0.532 (0.140, 0.920)	0.557 (0.140, 0.920)
SK^b_H	0.520 (0.140, 0.880)	0.543 (0.160, 0.880)	**0.702 (0.360, 0.960)**	**0.778 (0.420, 0.980)**
SK^b_w	0.743 (0.560, 0.900)	0.777 (0.600, 0.920)	0.565 (0.220, 0.880)	0.658 (0.320, 0.900)
SK^b_{un}	0.524 (0.220, 0.820)	0.515 (0.240, 0.800)	0.562 (0.260, 0.780)	0.472 (0.240, 0.740)
MK-BMC	0.762 (0.560, 0.920)	0.776 (0.540, 0.940)	0.653 (0.360, 0.880)	0.679 (0.360, 0.920)
Simulation Scenario II: not phylogenetically-related subset of OTUs				
Model (Association)	A (Abundance)	A (Abundance)	B (Presence/Absence)	B (Presence/Absence)
OTU proportion (number)	11.41% (9)	4.77% (9)	10.91% (9)	2.26% (9)
Oracle	0.810 (0.700, 0.900)	0.833 (0.740, 0.920)	0.680 (0.480, 0.940)	0.749 (0.620, 0.880)
Mdeep	0.600 (0.020, 1.000)	0.552 (0.000, 1.000)	0.480 (0.000, 1.000)	0.519 (0.000, 1.000)
RF	0.594 (0.420, 0.760)	0.587 (0.420, 0.760)	**0.592 (0.420, 0.740)**	0.596 (0.420, 0.760)
PAAM-RF	0.606 (0.440, 0.760)	0.572 (0.400, 0.740)	0.569 (0.420, 0.740)	0.543 (0.380, 0.720)
SK^b_{BC}	**0.795 (0.500, 0.980)**	**0.787 (0.360, 1.000)**	0.530 (0.140, 0.900)	0.595 (0.200, 0.940)
SK^b_H	0.404 (0.060, 0.800)	0.490 (0.140, 0.820)	0.542 (0.220, 0.800)	**0.771 (0.500, 0.940)**
SK^b_w	0.619 (0.320, 0.880)	0.683 (0.360, 0.940)	0.494 (0.180, 0.800)	0.542 (0.200, 0.860)
SK^b_{un}	0.480 (0.200, 0.780)	0.441 (0.160, 0.740)	0.357 (0.120, 0.620)	0.502 (0.240, 0.740)
MK-BMC	0.585 (0.220, 0.900)	0.596 (0.220, 0.880)	0.495 (0.200, 0.780)	0.720 (0.420, 0.920)
Simulation Scenario III: mixture of Scenario I and Senario II				
Association	$P_{phy} + P_{unphy}$	$P_{phy} + A_{unphy}$	$A_{phy} + P_{unphy}$	$A_{unphy} + P_{unphy}$
OTU proportion (number)	4.59%+10.91% (29+9)	1.43%+11.41% (25+9)	10.38%+2.26% (57+9)	11.41%+2.26% (9+9)
Oracle	0.882 (0.740, 0.980)	0.842 (0.740, 0.920)	0.840 (0.740, 0.940)	0.840 (0.740, 0.920)
Mdeep	0.508 (0.000, 1.000)	0.561 (0.000, 1.000)	0.560 (0.020, 0.980)	0.573 (0.000, 1.000)
RF	0.588 (0.420, 0.760)	0.592 (0.440, 0.760)	0.613 (0.440, 0.760)	0.600 (0.440, 0.760)
PAAM-RF	0.560 (0.400, 0.720)	0.620 (0.460, 0.780)	0.675 (0.500, 0.840)	0.585 (0.420, 0.740)
SK^b_{BC}	0.537 (0.140, 0.920)	**0.710 (0.320, 0.960)**	0.717 (0.440, 0.920)	**0.713 (0.320, 0.960)**
SK^b_H	**0.615 (0.30, 0.880)**	0.624 (0.220, 0.940)	0.695 (0.320, 0.940)	0.618 (0.240, 0.900)
SK^b_w	0.531 (0.220, 0.820)	0.625 (0.300, 0.860)	0.679 (0.480, 0.860)	0.580 (0.280, 0.840)
SK^b_{un}	0.411 (0.140, 0.700)	0.462 (0.200, 0.740)	0.495 (0.220, 0.760)	0.472 (0.200, 0.760)
MK-BMC	0.558 (0.260, 0.860)	0.643 (0.320, 0.920)	**0.726 (0.480, 0.920)**	0.630 (0.320, 0.880)

Table S10: AUC means and 0.025 and 0.975 quantiles (in parentheses) in test sets over 1000 simulations for data generated with $\beta = 3, n = 500$.

Simulation Scenario I: Signal OTUs are Phylogenetically-Related				
Model (Association)	A (Abundance)	A (Abundance)	B (Presence/Absence)	B (Presence/Absence)
OTU proportion (number)	10.38% (57)	4.91% (53)	4.59% (29)	1.43% (25)
Oracle	0.917 (0.892, 0.939)	0.911 (0.886, 0.935)	0.904 (0.881, 0.924)	0.907 (0.884, 0.929)
Mdeep	0.865 (0.828, 0.898)	0.847 (0.812, 0.883)	0.556 (0.503, 0.609)	0.587 (0.522, 0.651)
RF	0.804 (0.765, 0.842)	0.774 (0.731, 0.813)	0.761 (0.712, 0.804)	0.771 (0.723, 0.820)
PAAM-RF	0.887 (0.855, 0.916)	0.883 (0.853, 0.912)	0.756 (0.702, 0.804)	0.800 (0.759, 0.841)
SK^b_{BC}	0.794 (0.756, 0.832)	0.755 (0.713, 0.798)	0.611 (0.536, 0.684)	0.606 (0.520, 0.684)
SK^b_{H}	0.542 (0.484, 0.593)	0.569 (0.512, 0.627)	0.778 (0.730, 0.823)	0.785 (0.742, 0.833)
SK^b_{w}	**0.890 (0.861, 0.918)**	**0.893 (0.866, 0.919)**	0.674 (0.590, 0.744)	0.781 (0.739, 0.822)
SK^b_{un}	0.548 (0.492, 0.601)	0.574 (0.516, 0.633)	**0.794 (0.752, 0.833)**	**0.842 (0.797, 0.880)**
MK-BMC	**0.890 (0.861, 0.918)**	0.892 (0.866, 0.919)	**0.794 (0.751, 0.832)**	0.832 (0.792, 0.873)
Simulation Scenario II: Signal OTUs are Phylogenetically-Unrelated				
Model (Association)	A (Abundance)	A (Abundance)	B (Presence/Absence)	B (Presence/Absence)
OTU proportion (number)	11.41% (9)	4.77% (9)	10.91% (9)	2.26% (9)
Oracle	0.917 (0.896, 0.938)	0.911 (0.884, 0.936)	0.904 (0.880, 0.924)	0.910 (0.886, 0.932)
Mdeep	0.842 (0.804, 0.879)	0.814 (0.774, 0.855)	0.589 (0.526, 0.648)	0.603 (0.549, 0.658)
RF	0.825 (0.786, 0.862)	0.828 (0.787, 0.864)	0.831 (0.789, 0.867)	0.829 (0.789, 0.870)
PAAM-RF	0.820 (0.773, 0.862)	0.814 (0.765, 0.858)	0.809 (0.763, 0.854)	0.763 (0.707, 0.814)
SK^b_{BC}	**0.862 (0.816, 0.902)**	**0.868 (0.821, 0.908)**	0.668 (0.590, 0.729)	0.690 (0.607, 0.756)
SK^b_{H}	0.547 (0.491, 0.603)	0.630 (0.574, 0.685)	**0.839 (0.789, 0.884)**	**0.863 (0.805, 0.906)**
SK^b_{w}	0.807 (0.746, 0.856)	0.821 (0.747, 0.872)	0.614 (0.540, 0.688)	0.619 (0.539, 0.696)
SK^b_{un}	0.532 (0.481, 0.584)	0.612 (0.554, 0.668)	0.803 (0.756, 0.846)	0.810 (0.752, 0.862)
MK-BMC	0.842 (0.795, 0.885)	0.862 (0.809, 0.905)	0.837 (0.787, 0.884)	0.862 (0.803, 0.905)
Simulation Scenario III: Signal OTUs are a mixture of Scenarios I and II				
Association	$P_{phy} + P_{unphy}$	$P_{phy} + A_{unphy}$	$A_{phy} + P_{unphy}$	$A_{unphy} + P_{unphy}$
OTU proportion (number)	4.59%+10.91% (29+9)	1.43%+11.41% (25+9)	10.38%+2.26% (57+9)	11.41%+2.26% (9+9)
Oracle	0.946 (0.929, 0.963)	0.952 (0.934, 0.967)	0.952 (0.935, 0.967)	0.952 (0.933, 0.966)
Mdeep	0.570 (0.513, 0.623)	0.750 (0.700, 0.795)	0.755 (0.710, 0.798)	0.754 (0.709, 0.798)
RF	0.784 (0.738, 0.827)	0.790 (0.748, 0.833)	0.814 (0.772, 0.855)	0.820 (0.777, 0.861)
PAAM-RF	0.761 (0.707, 0.811)	0.803 (0.760, 0.844)	0.802 (0.759, 0.843)	0.766 (0.713, 0.816)
SK^b_{BC}	0.624 (0.550, 0.689)	0.751 (0.697, 0.803)	0.719 (0.672, 0.763)	0.758 (0.699, 0.808)
SK^b_{H}	0.803 (0.746, 0.857)	0.695 (0.645, 0.747)	0.755 (0.702, 0.810)	0.754 (0.698, 0.803)
SK^b_{w}	0.623 (0.546, 0.692)	0.771 (0.714, 0.818)	0.776 (0.734, 0.816)	0.710 (0.642, 0.770)
SK^b_{un}	0.790 (0.735, 0.843)	0.736 (0.689, 0.780)	0.717 (0.658, 0.772)	0.712 (0.656, 0.766)
MK-BMC	**0.813 (0.754, 0.866)**	**0.823 (0.773, 0.868)**	**0.817 (0.773, 0.858)**	**0.831 (0.769, 0.885)**

Table S11: Simulation results to investigate signal density with AUC means and 0.025 and 0.975 quantiles (in parentheses) in testing sets over 1,000 simulations.

	Signal OTUs are Phylogenetically-Related		Signal OTUs are Phylogenetically-Unrelated	
Model (Association)	A (Abundance)	A (Abundance)	A (Abundance)	A (Abundance)
Total Abundance (number)	4.59% (29)	4.91% (53)	4.77% (9)	4.84% (18)
Oracle	0.847 (0.814, 0.879)	0.849 (0.815, 0.881)	0.848 (0.814, 0.882)	0.849 (0.815, 0.882)
Mdeep	0.764 (0.719, 0.809)	0.777 (0.733, 0.820)	0.746 (0.693, 0.794)	0.715 (0.666, 0.763)
RF	0.755 (0.712, 0.798)	0.714 (0.670, 0.761)	0.756 (0.707, 0.799)	0.720 (0.667, 0.768)
PAAM-RF	0.790 (0.748, 0.832)	0.818 (0.783, 0.856)	0.743 (0.686, 0.794)	0.660 (0.602, 0.714)
SK_{BC}	0.759 (0.712, 0.803)	0.700 (0.655, 0.748)	0.719 (0.661, 0.772)	0.656 (0.597, 0.715)
SK_H	0.512 (0.440, 0.575)	0.518 (0.421, 0.593)	0.580 (0.411, 0.640)	0.575 (0.400, 0.646)
SK_w	0.770 (0.708, 0.820)	0.796 (0.749, 0.842)	0.656 (0.591, 0.719)	0.583 (0.382, 0.654)
SK_{un}	0.530 (0.430, 0.598)	0.524 (0.428, 0.592)	0.574 (0.442, 0.631)	0.558 (0.420, 0.622)
SK^b_{BC}	0.776 (0.731, 0.818)	0.717 (0.675, 0.762)	**0.798 (0.741, 0.848)**	**0.717 (0.255, 0.794)**
SK^b_H	0.537 (0.480, 0.595)	0.550 (0.491, 0.604)	0.601 (0.540, 0.661)	0.595 (0.541, 0.648)
SK^b_w	**0.796 (0.752, 0.838)**	**0.832 (0.796, 0.866)**	0.755 (0.680, 0.815)	0.660 (0.577, 0.726)
SK^b_{un}	0.554 (0.493, 0.611)	0.554 (0.501, 0.609)	0.586 (0.529, 0.643)	0.573 (0.513, 0.630)
MK-BMC	0.791 (0.746, 0.831)	0.824 (0.784, 0.861)	0.783 (0.720, 0.838)	0.697 (0.625, 0.765)

Table S12: Simulation results to investigate boosting weights in distance measures with AUC means and 0.025 and 0.975 quantiles (in parentheses) in testing sets over 1000 simulations for data generated with 57 abundant (10.38%) phylogenetically-related signal OTUs.

training sample size	500	300	200	100
Oracle	0.858 (0.825, 0.886)	0.858 (0.814, 0.895)	0.858 (0.808, 0.904)	0.857 (0.782, 0.920)
Mdeep	0.797 (0.752, 0.839)	0.787 (0.729, 0.840)	0.778 (0.710, 0.840)	0.744 (0.623, 0.842)
RF	0.747 (0.703, 0.792)	0.722 (0.663, 0.784)	0.699 (0.614, 0.774)	0.653 (0.531, 0.770)
PAAM-RF	0.825 (0.787, 0.863)	0.819 (0.768, 0.866)	0.814 (0.753, 0.869)	0.795 (0.701, 0.876)
SK_{BC}	0.762 (0.723, 0.805)	0.754 (0.695, 0.806)	0.744 (0.672, 0.808)	0.710 (0.522, 0.823)
SK_{H}	0.513 (0.429, 0.585)	0.504 (0.423, 0.580)	0.504 (0.412, 0.592)	0.501 (0.383, 0.614)
SK_{w}	0.814 (0.772, 0.853)	0.809 (0.748, 0.858)	0.802 (0.735, 0.862)	0.783 (0.682, 0.875)
SK_{un}	0.515 (0.434, 0.583)	0.506 (0.421, 0.582)	0.503 (0.402, 0.595)	0.500 (0.390, 0.617)
SK^b_{BC} (pvalues)	0.755 (0.716, 0.797)	0.756 (0.702, 0.807)	0.755 (0.685, 0.816)	0.746 (0.643, 0.836)
SK^b_{H} (pvalues)	0.532 (0.482, 0.585)	0.522 (0.450, 0.592)	0.519 (0.439, 0.602)	0.513 (0.395, 0.628)
SK^b_{w} (pvalues)	**0.834 (0.799, 0.869)**	**0.834 (0.787, 0.876)**	**0.833 (0.777, 0.883)**	**0.827 (0.744, 0.904)**
SK^b_{un} (pvalues)	0.537 (0.485, 0.590)	0.527 (0.455, 0.592)	0.522 (0.449, 0.605)	0.513 (0.402, 0.620)
MK-BMC (pvalues)	**0.834 (0.798, 0.869)**	0.824 (0.771, 0.872)	0.814 (0.752, 0.869)	0.802 (0.711, 0.883)
SK^b_{BC} (coefficient)	0.773 (0.734, 0.815)	0.765 (0.708, 0.818)	0.750 (0.681, 0.813)	0.697 (0.573, 0.802)
SK^b_{H} (coefficients)	0.530 (0.480, 0.583)	0.521 (0.455, 0.588)	0.518 (0.439, 0.597)	0.512 (0.396, 0.628)
SK^b_{w} (coefficients)	**0.834 (0.798, 0.869)**	0.831 (0.782, 0.874)	0.825 (0.766, 0.877)	0.797 (0.696, 0.878)
SK^b_{un} (coefficients)	0.533 (0.482, 0.589)	0.524 (0.453, 0.589)	0.520 (0.444, 0.601)	0.512 (0.407, 0.630)
MK-BMC (coefficients)	0.818 (0.781, 0.855)	0.804 (0.750, 0.851)	0.780 (0.703, 0.844)	0.704 (0.566, 0.817)

Table S13: Summary of the 3 outcomes together with AUC means and 0.025 and 0.975 quantiles (in parentheses) in testing sets across 1,000 50/50 random splits when age information is not used.

	Thyroid	Obesity	IBD
	gut	gut	gut
#samples	3131	1994	3097
#cases (%)	378 (12.1%)	357 (17.9%)	140 (4.5%)
#OTUs	13359	11992	13242
Mdeep	0.569 (0.534, 0.601)	0.601 (0.561, 0.637)	0.599 (0.537, 0.653)
RF	0.561 (0.528, 0.593)	0.597 (0.559, 0.636)	0.652 (0.602, 0.697)
PAAM-RF	0.583 (0.550, 0.615)	**0.649 (0.611, 0.688)**	0.660 (0.613, 0.704)
SK^b_{BC}	0.597 (0.562, 0.629)	0.614 (0.567, 0.659)	0.648 (0.579, 0.708)
SK^b_H	0.603 (0.567, 0.636)	0.648 (0.603, 0.693)	0.669 (0.614, 0.720)
SK^b_w	0.571 (0.523, 0.613)	0.604 (0.542, 0.657)	0.628 (0.561, 0.692)
SK^b_{un}	**0.607 (0.568, 0.644)**	0.647 (0.601, 0.690)	0.674 (0.613, 0.726)
MK-BMC	0.606 (0.565, 0.642)	**0.649 (0.604, 0.696)**	**0.676 (0.616, 0.731)**

Table S14: Body habitat distribution of the 4620 USA samples with OTU total counts $\geq$ 1,250.

	Ear	Eye	Feces	Hair	Nose	Oral cavity	Skin	Vagina
Count (percentage)	23 (0.50%)	26 (0.56%)	4103 (88.81%)	1 (0.02%)	28 (0.61%)	212 (4.59%)	214 (4.63%)	13 (0.28%)

Table S15: Variable names and responses in the online sample sheet for the 3 outcomes.

Outcome	Value name	Response for case samples	Response for control samples
Thyroid	thyroid	Diagnosed by a medical professional (doctor, physician assistant), Diagnosed by an alternative medicine practitioner, Self-diagnosed	I do not have this condition
Obesity	bmi_corrected	≥30	<25&≥18.5
IBD	ibd	Diagnosed by a medical professional (doctor, physician assistant), Diagnosed by an alternative medicine practitioner, Self-diagnosed	I do not have this condition

Table S16: Summary statistics of the 3 binary outcomes for gut microbiome samples where covariate information (age) is utilized for sample filtering and where it is not.

	Thyroid	Obesity	IBD
	gut	gut	gut
covariate information is not used for sample filtering			
Sample size	3131	1994	3097
Number of cases (%)	378 (12.1%)	357 (17.9%)	140 (4.5%)
Number of OTUs	13359	11992	13242
covariate information is used for sample filtering			
Sample size	2941	1930	2913
Number of cases (%)	355 (12.1%)	348 (18.0%)	136 (4.7%)
Number of OTUs	13115	11841	13028

Supplementary Material References

[1] J Gregory Caporaso, Justin Kuczynski, Jesse Stombaugh, Kyle Bittinger, Frederic D Bushman, Elizabeth K Costello, Noah Fierer, Antonio Gonzalez Peña, Julia K Goodrich, Jeffrey I Gordon, et al. Qiime allows analysis of high-throughput community sequencing data. *Nature methods*, 7(5):335–336, 2010.

[2] Stilianos Louca and Michael Doebeli. Efficient comparative phylogenetics on large trees. *Bioinformatics*, 34(6):1053–1055, 2018.

[3] Daniel McDonald, Embriette Hyde, Justine W Debelius, James T Morton, Antonio Gonzalez, Gail Ackermann, Alexander A Aksenov, Bahar Behsaz, Caitriona Brennan, and Yingfeng Chen. American gut: an open platform for citizen science microbiome research. *mSystems*, 3(3), 2018.

[4] Daniel McDonald, Morgan N Price, Julia Goodrich, Eric P Nawrocki, Todd Z DeSantis, Alexander Probst, Gary L Andersen, Rob Knight, and Philip Hugenholtz. An improved greengenes taxonomy with explicit ranks for ecological and evolutionary analyses of bacteria and archaea. *The ISME journal*, 6(3):610–618, 2012.
